# Exploration of the relationship between hippocampus and immune system in schizophrenia based on immune infiltration analysis

**DOI:** 10.3389/fimmu.2022.878997

**Published:** 2022-08-02

**Authors:** Yanhong Du, Yao Gao, Guangxian Wu, Zexuan Li, Xinzhe Du, Junxia Li, Xinrong Li, Zhifen Liu, Yong Xu, Sha Liu

**Affiliations:** ^1^ Department of Psychiatry, First Hospital/First Clinical Medical College of Shanxi Medical University, Taiyuan, China; ^2^ Shanxi Key Laboratory of Artificial Intelligence Assisted Diagnosis and Treatment for Mental Disorder, First Hospital of Shanxi Medical University, Taiyuan, China; ^3^ Department of Physiology, School of Basic Medical Sciences, Shanxi Medical University, Taiyuan, China; ^4^ Department of Mental Health, Shanxi Medical University, Taiyuan, China

**Keywords:** schizophrenia hippocampus, immune infiltration, immune-related genes, immune pathways, bioinformatics

## Abstract

Immune dysfunction has been implicated in the pathogenesis of schizophrenia (SZ). Despite previous studies showing a broad link between immune dysregulation and the central nervous system of SZ, the exact relationship has not been completely elucidated. With immune infiltration analysis as an entry point, this study aimed to explore the relationship between schizophrenia and the immune system in more detail from brain regions, immune cells, genes, and pathways. Here, we comprehensively analyzed the hippocampus (HPC), prefrontal cortex (PFC), and striatum (STR) between SZ and control groups. Differentially expressed genes (DEGs) and functional enrichment analysis showed that three brain regions were closely related to the immune system. Compared with PFC and STR, there were 20 immune-related genes (IRGs) and 42 immune pathways in HPC. The results of immune infiltration analysis showed that the differential immune cells in HPC were effector memory T (Tem) cells. The correlation of immune-related DEGs (IDEGs) and immune cells further analysis showed that *NPY*, *BLNK*, *OXTR*, and *FGF12*, were moderately correlated with Tem cells. Functional pathway analysis indicated that these four genes might affect Tem by regulating the PI3K-AKT pathway and the neuroactive ligand-receptor interaction pathway. The receiver operating characteristic curve (ROC) analysis results indicated that these four genes had a high diagnostic ability (AUC=95.19%). Finally, the disease animal model was successfully replicated, and further validation was conducted using the real-time PCR and the western blot. These results showed that these gene expression changes were consistent with our previous expression profiling. In conclusion, our findings suggested that HPC in SZ may be more closely related to immune disorders and modulate immune function through Tem, PI3K-Akt pathway, and neuroactive ligand-binding receptor interactions. To the best of our knowledge, the Immucell AI tool has been applied for the first time to analyze immune infiltration in SZ, contributing to a better understanding of the role of immune dysfunction in SZ from a new perspective.

## 1 Introduction

Schizophrenia (SZ) is thought to be a devastating and persistent psychotic illness. There are roughly 1% of the world’s population afflicted, and the number of afflicted populations is increasing (1). The clinical symptoms of SZ are complex and diverse, mainly manifested as positive symptoms, negative symptoms, and cognitive dysfunction (2, 3). These symptoms markedly affect the patients’ quality of life and social functioning. Meanwhile, this undoubtedly increases the burden on the social and family (4). However, the precise pathogenesis of SZ is not elucidated yet (5).

Currently, it is generally accepted that the pathogenesis of SZ is the comprehensive result of inherited and environmental factors, involving multiple systems (5). Thus, several hypotheses have been advanced to explain the etiology of SZ, including the monoamine neurotransmitter hypothesis (6, 7), the neuroendocrine hypothesis (8), the immunoinflammatory hypothesis (9), and so on. Accumulating evidence supports that immune dysfunction plays an essential role in SZ. Simarily, this point is also supported by epidemiological studies (9). Epidemiological and animal studies consistently find that prenatal infection can increase SZ risk of offspring, and the central nervous system (CNS) infections during earlier life stages also increase the risk of adults (10, 11), both having lasting effects on the neuroimmune system (12, 13). And, genome-wide association study of SZ also supports the link between the immune system and SZ (14).

When immune dysregulation occurs in CNS, the resident immune cells, microglia as well as astrocyte, are aberrantly activated, releasing inflammatory mediators, and recruiting other types of immune cells (15, 16). It may even cause secondary neuropathological alteration (17), affecting multiple brain areas such as the hippocampus (HPC), prefrontal cortex (PFC), and striatum (STR). Compared with other cortical areas, HPC has unique characteristics in structure, function, and plasticity potential (18). Meanwhile, HPC has the most significant association with the pathophysiology of SZ (19, 20), and it is also among the main focus points of brain areas in the study of mental diseases. However, the exact mechanism of neuroimmune dysregulation has not been well determined in specific brain areas of SZ.

Considering that the normal function of the immune system is inseparable from the maintenance of the homeostasis of different types of immune cells. Therefore, it is necessary to clarify the composition of immune cells in the tissue microenvironment, and then to clarify which immune cells play an important role in the occurrence and development of diseases. To address this, there are many tools for assessing immune infiltration, and each has its advantages. Compared with other tools, Immune Cell Abundance Identifier (ImmuCellAI) can be used for immune infiltration analysis. This tool more precisely evaluates the abundance of numerous T lymphocyte subpopulations as well as other immune cells, identifying the difference of infiltrating immune cells in different groups, and so on (21). Nowadays, ImmuCellAI can be used not only for tumors but also for immune-infiltration analysis of non-tumor diseases such as Alzheimer’s disease (22), depression (23), carotid artery atherosclerosis (24), and so on. Collectively, using the ImmuCellAI tool is also feasible to analyze immune cells in SZ.

**Figure 1:**
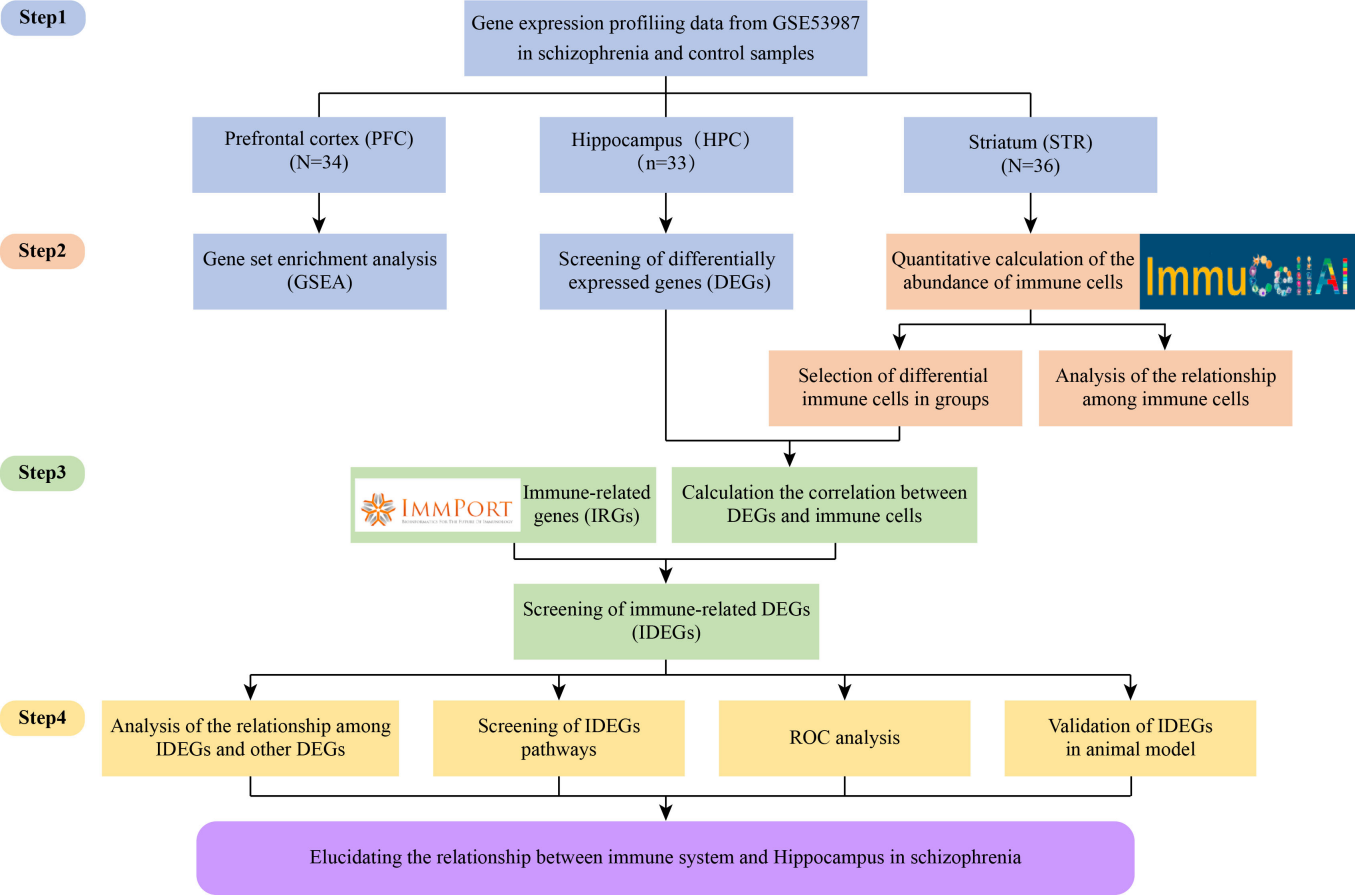
Flow chart of the whole research design.

Our study aimed to investigate the relationship between immunity dysfunction and SZ from brain regions, immune cells, immune-related genes (IRGs), and functional pathways. First, this study identified differentially expressed genes (DEGs) in SZ and control groups in HPC, PFC, and STR. And gene set enrichment analysis (GSEA) was performed to identify functional pathways. Second, the ImmuCellAI tool was used for analyzing the different immune infiltration patterns and determining the differential immune cells in SZ and control groups in three brain regions. Third, the correlation were evaluated between DEGs and differentially expressed immune cells. And according to the IRGs from the ImmPort database, immune-related differentially expressed genes (IDEGs) were screened. Moreover, the enrichment pathway analysis and the evaluation of the diagnostic ability were performed on the candidate IDEGs. Finally, the receiver operating characteristic (ROC) curve of IDEGs was performed to assess diagnostic ability. Candidate IDEGs were validated in the animal model. Meanwhile, to clarify the study project more clearly, the flowchart is displayed in **Figure 1**.

## 2 Methods and materials

### 2.1 Acquisition and processing of gene expression array data

The data of GSE53987, acquired from the Gene Expression Omnibus (GEO) database, contains information on postmortem brain tissue samples from 19 patients with SZ and 19 matched healthy controls. These brain tissue samples were obtained from the Allegheny County Medical Examiner’s Office (Pittsburgh, PA) and approved by the University of Pittsburgh’s Committee for the Oversight of Research and Clinical Trials Involving the Dead and Institutional Review Board for Biomedical Research (25). There were no significant differences in gender and mean age among these subjects (25, 26). In the present study, we selected HPC (15 SZ and 18 control), PFC (15 SZ and 19 control), and STR (18 SZ and 18 control) from SZ and control groups for subsequent analysis. And there were no significantly different in postmortem interval (PMI) and brain pH (26), see **Supplementary Table 1**. Subsequently, the GSE53987 expression matrix was combined with the platform of GPL570 ([HG-U133_Plus_2] Affymetrix Human Genome U133 Plus 2.0 Array), which enabled the probes to be transformed into matched gene symbols (26). When a gene with multiple probe expression data, the median expression value was calculated as the gene expression level. Then these sample data were normalized by quantile normalization. And the GSE17612 dataset was used as an independent external validation dataset, which includes 28 patients with SZ and 23 healthy controls. These brain tissue samples were obtained from the Imperial College London and received from the West London Mental Health Ethical Research Committee (27). And there were no significant differences in age and PMI in SZ and control groups, see **Supplementary Table 1**. Moreover, GSE17612 also needs to do the same data processing.

### 2.2 Screening of DEGs in three brain regions and GSEA

The DEGs in three brain regions were analyzed *via* the SangerBox website^1^ and DEGs were screened based on a criterion (*P*< 0.05 and fold change≥ 1.5). Then volcano plots and heatmaps were visualized using pheatmap (28) and ggplot2 (29) packets of R language.

To perform GSEA analysis for all differently expressed genes in SZ and control groups, we used GSEA *via* the Web-based Gene Set Analysis Toolkit^2^ (WebGestalt) (30). The Kyoto Encyclopedia of Genes and Genomes (KEGG) and Reactome databases are used to analyze the gene enrichment pathway and identify functional enrichment pathways of IDEGs. When |Normalized Enrichment Score |(NES)|> 1, *P*< 0.05 and the false discovery rate (FDR)< 0.25 were utilized to select meaningful pathways.

### 2.3 Immune infiltration analysis and correlation analysis

To explore the immune infiltration in SZ, we used ImmuCellAI^3^ (21) to predict the abundance of 24 immune-cell phenotypes in these samples by uploading the expression matrix data of GSE53987. Subsequently, the Wilcoxon rank-sum test was used to evaluate the differential immune cells in two groups. And the stats (31) and vioplot pockets of the R package were used for visualization. *P*< 0.05 was considered to be statistically significant. Meanwhile, we also used the xCell^4^ algorithm to infer immune infiltration.

Spearman correlation analysis was performed for investigating the relationship between immune cells, the correlation of DEGs and immune cells. Meanwhile, |Correlation Coefficient|>  0.5 and *P* < 0.05 were set as the criteria, and the outcomes were visualized basing the “ggplot2” package (32).

### 2.4 Identifying and comprehensive analysis of IDEGs

#### 2.4.1 Identifying IDEGs

To screen out which DEGs are closely related to specific immune cells, we first screened DEGs with a relatively strong correlation with specific immune cells basing the outcomes of correlation analysis. Subsequently, DEGs of the three brain regions and IRGs were crossed using the Venn-diagram packet of R software (33). These IRGs were retrieved from the Immport Shared Data^5^. And overlapping IRGs from DEGs were selected for further analysis.

#### 2.4.2 Constructing the protein-protein interaction network

To determine functional interactions relationships among the proteins, the STRING database^6^ (34) was applied to confirm DEGs’ relationship and search for the interactions among proteins. And protein-protein interaction (PPI) networks of three brain regions were constructed using the PPI pairs meeting interaction scores> 0.4. Then, PPI networks were built and visualized *via* Cytoscape v.3.7.2 (35).

#### 2.4.3 Analysis of functional enrichment pathway

To further investigate the function of IDEGs, the pathway data from the Database for Annotation, Visualization and Integrated Discovery (DAVID) v6.8 database^7^ was applied for the Kyoto Encyclopedia of Genes and Genomes (KEGG) pathway analysis of IDEGs and other DEGs based on the interaction between IDEGs and other DEGs. The *P*-value was set to 0.05 as the cutoff value.

#### 2.4.4 ROC analysis

ROC curves were formulated, and the area under the ROC curve (AUC) was calculated to evaluate the diagnostic abilities of IDEGs (36). The ROC curves were plotted by the OmicStudio tools^8^.

### 2.5 Animal experimental validation

To validate the results of the above screening, we used MK-801 for establishing an animal model of SZ for further validation. Detailed descriptions of experimental methods and statistical analyses are provided in the supplementary information.

The experimental subjects were 16 male Sprague-Dawley rats (Beijing Vital River Laboratory Animal Technology Co., Ltd., Beijing, China). Under controlled laboratory conditions with adequate food and water. The animals were divided into two groups, and the rats were acclimated to the laboratory environment for seven days before the experiments. At four weeks of age, 0.5 mg/kg the non-competitive Anti-N-methyl-D-aspartate (NMDA) antagonist (MK-801, Sigma-Aldrich, USA) and the equal volume of 5mL/kg saline (0.9% NaCl) were injected intraperitoneally (i.p.) for 14 consecutive days, respectively.

To validate the success of the animal model of SZ, we used behavioral testing (open field test, novel object recognition test, and Y-maze test). Next, to further validate the related immune genes. We employed real-time quantitative reverse transcription-polymerase chain reaction (RT-qPCR) and Western blotting. RT-qPCR and Western Blotting were analyzed with an Unpaired two-tailed t-test. Normality and homogeneity of variance were verified using the Shapiro–Wilk test and Levene’s test, respectively. Data are described by means ± SEMs. For all statistical tests, *P*< 0.05 was considered statistically significant.

## 3 Results

### 3.1 Screening of DEGs and GSEA analysis in three brain regions

#### 3.1.1 Screening of DEGs

**Figure 2:**
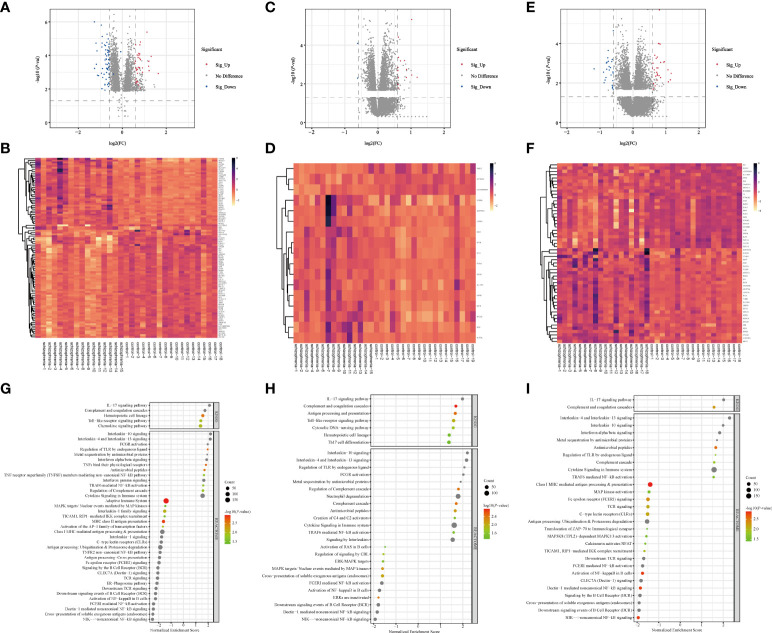
Screening of DEGs and GSEA analysis in three brain regions **(A–F)** Volcano plots and heatmap of DEGs in three brain regions. The horizontal axis represents the log2 (Fold Change), and the vertical axis represents the -log10(P-value) gene expression. In volcano plots, the red dots represent significantly up-regulated DEGs, the blue dots represent down-regulated DEGs, and the grey dots represent genes with |FC| < 1.5 and/or P > 0.05. In heatmaps, the diagram presents the result of the hierarchical clustering of DEGs, and each row represents a DEG and each column, a sample. The color scale illustrates the relative level of DEG expression: red, below the reference channel; green, higher than the reference. **(A, B)** HPC. **(C, D)** PFC. **(E, F)** STR. HPC, hippocampus; PFC, prefrontal cortex; STR, striatum. **(G–I)** Bubble plots of immune-related signaling pathways. Reactome (2016) and KEGG (2019) datasets analyzed immune system pathways using GSEA analysis. The significant function pathways involved with the immune system were presented in different brain areas, respectively. P < 0.05, FDR < 0.25, and |NES| > 1 was considered statistically significant. The bigger triangles, the greater the leading edge number. **(A)** HPC. **(B)** PFC. **(C)** STR. KEGG, Kyoto Encyclopedia of Genes and Genomes; GSEA, Gene Set Enrichment Analysis; FDR, false discovery rate; NES, normalized enrichment score; HPC, hippocampus; PFC, prefrontal cortex; STR, striatum.

Altogether, there were 172 DEGs in three brain regions from SZ and control groups, in which there were 100 DEGs (39 up-regulated DEGs, 61 down-regulated DEGs) from HPC, 17 DEGs (14 up-regulated DEGs, 3 down-regulated DEGs) from PFC, and 55 DEGs (29 up-regulated DEGs, 26 down-regulated DEGs) from STR (**Supplementary Tables 3–5**). The volcano plots and clustering heatmaps all showed the differential distribution of gene expression levels in SZ and control groups according to different brain regions (**Figure 2**). By performing the chip (GSE17612) analysis, there were *HILPDA*, *S100A8*, *S100A9*, *HSPB1*, and *BAG3*, having statistically significant differences (*P <*0.05) between the SZ and control groups (**Supplementary Figure 1**). This result is consistent with the analysis results of the brain region (PFC) in the chip (GSE53987) we used, which also demonstrates the reliability of the method.

The number of DEGs in different brain regions was distinct. Seven DEGs were shared by the HPC, PFC, and STR, including *S100A8*, *APOLD1*, *ADM*, *HILPDA*, *DDIT4*, *HSPB1*, and *BAG3*. And these genes were up-regulated DEGs. According to our analysis results, the changing trend of the shared DEGs in different brain regions in SZ is consistent, compared with the control group. But these shared genes fold changes in the three brain regions were different (**Supplementary Table 6**). The probable explanation for this is that the effect is even more significant for HPC in SZ compared with the other two brain regions. And we found that the number of IRGs in HPC, PFC, and STR was 20, 4, and 9. From these data, we can see that the number of DEGs are different in different brain tissues, and there is the largest number of DEGs in HPC.

#### 3.1.2 GSEA analysis

To analyze the function of the genes, we performed GSEA analysis on three brain regions using the online website of WebGestalt. The immune system pathways were screened. All statistically significant immune pathways in three brain regions from the Reactome pathway database and the KEGG database are displayed in **Figure 2G–I** and **Supplementary Tables 7–9**. Then pathways were ordered according to the absolute value of the Normalized Enrichment Score (NES). In HPC, there are 42 immune-related pathways, the top three immune pathways including the non-canonical NF-kB signaling pathway, cross-presentation of soluble exogenous antigens (endosomes) pathway, and Dectin-1 mediated non-canonical NF-κB signaling pathway. In PFC, there are 32 immune-related pathways, the top three immune pathways including the Interleukin-10 signaling pathway, Interleukin-4 and Interleukin-13 signaling pathway, and regulation of TLR by endogenous ligand pathway. In STR, there are 30 immune-related pathways, the top three immune pathways including the Interleukin-4 and Interleukin-13 signaling pathway, IL-17 signaling pathway, and Interleukin-10 signaling pathway. In terms of this result, we found the enriched immune system pathway in HPC is also the most numerous, compared with the other two brain regions. And these enriched immune system pathways exist in three brain regions, which also demonstrated dysregulated immune system pathways in SZ and provided a base for our subsequent analysis.

### 3.2 Immune infiltration analysis

#### 3.2.1 The relative abundances analysis of immune cells

**Figure 3:**
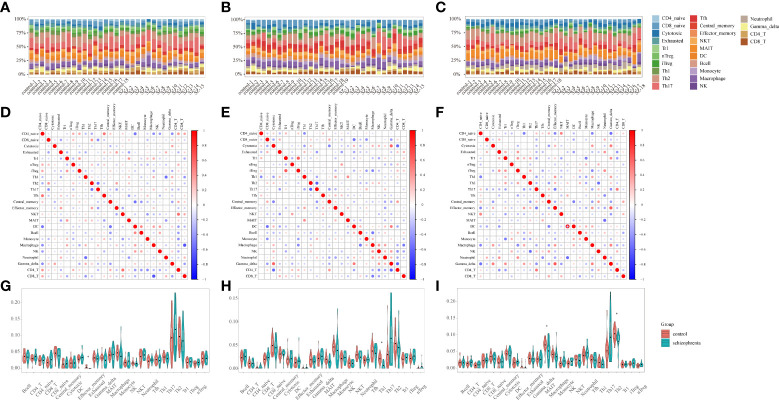
Immune infiltration analysis **(A-C)** Stacked bar plots showing the proportion of each 24 subtypes of immune cells. Each stacked bar plot corresponds to one immune cell. **(A)** HPC. **(B)** PFC. **(C)** STR. HPC, hippocampus; PFC, prefrontal cortex; STR, striatum. **(D–F)** The correlation matrix diagram of 24 immune-cell subtypes. The red color represents a high positive correlation, and the blue indicates a high negative correlation. The larger the circle, the higher the correlation coefficient. The correlation coefficients were computed using the Spearman correlation coefficient. **(D)** HPC. **(E)** PFC. **(F)** STR. HPC, hippocampus; PFC, prefrontal cortex; STR, striatum. **(G-I)** Violin plots of the abundance of 24 immune-cell subtypes between schizophrenia and control groups. The red and blue bars represent the SZ and control groups, respectively. **(G)** HPC. **(H)** PFC. **(I)** STR. *P < 0.05 vs. control group. HPC, hippocampus; PFC, prefrontal cortex; STR, striatum.

Considering that immune response possesses an essential function in SZ, the ImmuCellAI tool was used to evaluate the abundance of immune cells in three brain tissues and the difference of infiltrating immune cells between SZ and control groups. The abundance of 24 immune-cell subtypes was analyzed for each sample (**Figures 3A–C**). It can be seen that the proportion of immune cells is different in three brain regions, which further suggests that it is highly warranted to further analysis in the three brain regions.

#### 3.2.2 Correlation analysis between immune cells

The relationships among 24 immune-cell subtypes are shown in **Figures 3D–F**. In HPC, macrophages cells were negatively correlated with CD8+ T cells (*r*= -0.68, *P*= 0.004) in SZ and control groups. In PFC, Type 1 regulatory T (Trl) cells had positively associated with induced regulatory T (iTregs) cells (*r*= 0.66, *P*= 0.007); in contrast, Th2 cells had inversely associated with T-helper 17 (Th17) cells (*r*= -0.62, *P*= 0.01) in SZ and control groups. In STR, Tem cells had positively correlated with gamma delta T cells (*r*= 0.64, *P*= 0.006) in SZ and control groups. The correlation coefficients of other immune cell types are all below 0.62. The results suggested that interacting immune-cell subtypes were different in three brain regions. There might be a synergistic or antagonistic relationship between these interacting immune cells.

#### 3.2.3 Identifying differential immune cells in three brain regions

By identifying differential immune cells in three brain regions between SZ and control groups, there are effector memory T (Tem) in HPC, T-helper 1 (Th1) cells in PFC, T-helper 2 (Th2) cells as well as mucosal-associated invariant T (MAIT) cells in STR (**Figures 3G–I**). In the SZ group, the abundance of Tem cells in HPC, Th1 cells in PFC, Th2 cells, and MAIT cells in STR are generally lower than in the control group. The results suggested that the three brain regions harbored different immune-cell subtypes, and these different immune cells belonged to T cells.

Furthermore, we uploaded the gene expression data to Xcell to calculate the infiltration of 64 immune cells. For immune cell types shared by Xcell and ImmuCellAI tools, there are B-cells, CD4+ T-cells, CD4+ Tcm, CD4+ Tem, CD4+ memory T-cells, CD4+ naive T-cells, CD8+ T-cells, CD8+ Tcm, CD8+ Tem, CD8+ naive T-cells, DC, Macrophages, Monocytes, NK cells, NKT, Neutrophils, Tgd cells, Th1 cells, Th2 cells, and Tregs. In HPC, mostly shared immune cells are consistent with our results, except for Tgd (gamma delta T) cells, Th1 cells, and Tem cells (**Supplementary Figure 2A**). In PFC, mostly shared immune cells are consistent with our results, except for CD4+ naive T-cells (**Supplementary Figure 2B**). In STR, mostly shared by immune cells are consistent with our results, except for Th2 cells (**Supplementary Figure 2C**).

### 3.3 Correlation analysis of DEGs and immune cells in three brain region

#### 3.3.1 Correlation analysis of DEGs and 24 immune-cell subtypes

**Figure 4:**
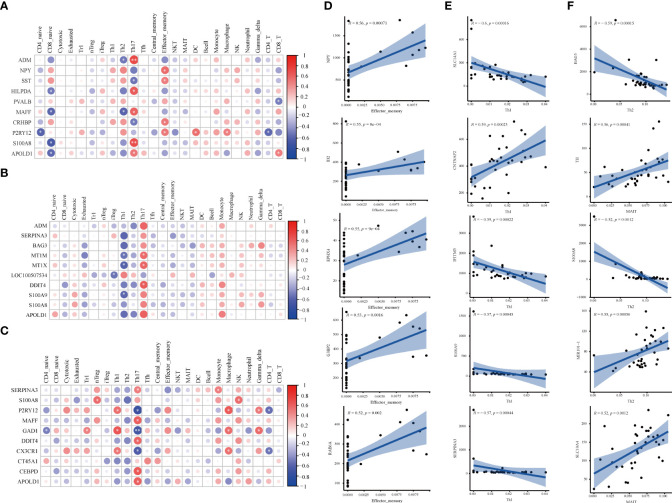
Correlation analysis of DEGs and immune cells in three brain regions. **(A-C)** The matrix heatmaps of the DEGs (top ten |FC|-value) and infiltrating immune cells. The size of the colored circles represents the statistical significance; the red circles represent a positive correlation, and the blue circles represent a negative correlation. The darker the color, the higher the correlation. *P< 0.05, **P< 0.001,* and ** are marked with white color. **(A)** HPC. **(B)** PFC. **(C)** STR. HPC, hippocampus; PFC, prefrontal cortex; STR, striatum. **(D–F)** The correlation plots between DEGs (top five |correlation coefficient|-value) and differential immune cells. Analysis was performed by Spearman correlation. R, Spearman coefficient. The blue line represents the linear prediction. The blue belt is the 95% confidence interval; points are the value of DEGs in all samples. |correlation coefficient| ≥ 0.5, P < 0.05 were considered statistically significant. DEGs, differentially expressed genes; FC, Fold Change; HPC, hippocampus; PFC, prefrontal cortex; STR, striatum.

The correlation between DEGs and 24 immune cells was performed *via* Spearman correlation analysis to screen DEGs related to differential immune cells. **Figures 4A–C** and **Supplementary Figure 3** show the results from the correlation analysis in HPC, PFC, and STR. In HPC, most DEGs are positively associated with Th17 and Tem, and negatively associated with Naïve CD8+ T cells; among the ten DEGs, *P2RY12* are positively associated with Tem, DC cells, and Macrophage, and negatively associated with Naïve CD4+ T and CD4+ T cells (**Figure 4A**). In PFC, most DEGs are strongly associated with Th17 and negatively associated with Th1 (**Figure 4B**). In STR, Th17 is strongly associated with most DEGs and strongly negatively associated with *GAD1* (**Figure 4C**). Surprisingly, these DEGs in the three brain regions are highly related to Th17.

#### 3.3.2 Correlation analysis of DEGs and differential immune cells

To screen DEGs related to differential immune cells, the correlation of DEGs and different immune cells was performed *via* Spearman correlation analysis. **Figures 4D–F** and **Supplementary Figure 4** show the results from the correlation analysis in HPC, PFC, and STR. In HPC, eleven DEGs related to Tem cells were screened, including *EPHX4* (*r*= 0.55, *P*= 0.025), *G3BP2* (*r*= 0.53, *P*= 0.035), *ID2* (*r*= 0.55, *P*= 0.022), *NPY* (*r*= 0.56, *P*= 0.020), and *RAB6A* (*r* =0.52, *P*= 0.040), (**Figure 4D**). In PFC, there were ten DEGs related to Th1 cells were screened, including *CNTNAP2* (*r*= 0.59, *P*= 0.011), *IFITM3* (*r*= -0.59, *P*= 0.011), *S100A9* (*r*=- 0.57, *P*= 0.015), *SERPINA3* (*r*= -0.57, *P*= 0.015), and *SLC14A1* (*r*= -0.60, *P*= 0.011) (**Figure 4E**). In STR, more total of seven DEGs related to differentiated immune cells were screened (**Figure 4F**). Five DEGs related to Th2 cells, *MIR101-1* (*r*= 0.55, *P*=0.012), *BAG3* (*r*= -0.59, *P*= 0.005), and *S100A8* (*r*= -0.52, *P*= 0.012), respectively; there was *TH* (*r*= 0.59, *P*= 0.0109), *SLC10A4* (*r*= 0.52, *P*= 0.020) being related to MAIT cells. The results imply that the above-mentioned genes might affect the activation of differential immune cells.

### 3.4 Identifying and comprehensive analysis of IDEGs

#### 3.4.1 Identifying of IDEGs

**Figure 5:**
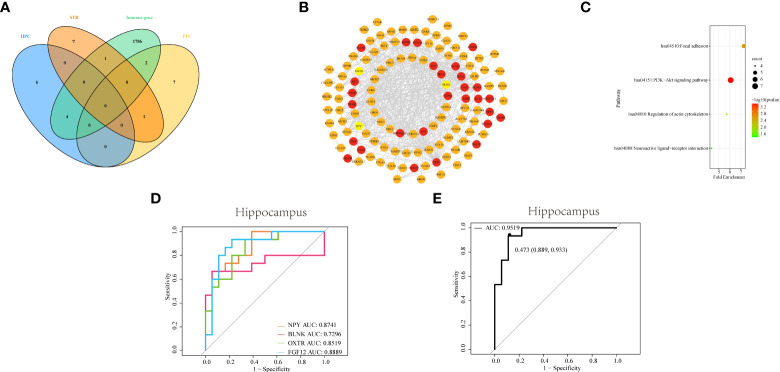
Identifying and comprehensive analysis of IDEGs **(A)** Venn diagram of DEGs with differential immune cells from three brain tissues and IRGs. **(B)** The construction of the PPI network and analysis of the relationship among IDEGs and other DEGs in the hippocampus. The yellow color of the circle represented proteins coded by IDEGs; the red color represented proteins coded by genes interacting with IDEGs. **(C)** Bubble plots of functional signaling pathways for IDEGs interacting with other DEGs. The color of dots represents -log(P-value), the size of the dots represents the number of enriched genes. P< 0.05 was considered statistically significant. DEGs, differentially expressed genes; IDEGs, immune-related differentially expressed genes. **(D–E)** ROC curves analysis. **(D)** ROC curve of NPY, BLNK, OXTR, and FGF12 in HPC. The AUC of NPY = 0.8741; the area under ROC curve of BLNK = 0.7295; the AUC of OXTR = 0.8519; the AUC of FGF12 = 0.8889. **(E)** ROC curve of the four-gene signature in HPC. The AUC = 0.9519 (95% confidence interval: 0.889-0.933).

To screen for DEGs related to immunity, we downloaded IRGs from the Immport database, and DEGs related to differential immune cells in three brain regions were intersected with IRGs, respectively. The Venn diagram showed that a total of 8 IDEGs were screened (**Figure 5A**). In HPC, genes common to IRGs and DEGs contain *NPY*, *BLNK*, *OXTR*, and *FGF12*. There were *SERPINA3* and *S100A* in PFC, and there was only *S100A8* in STR were eligible for screening. From this result, we can see that there is the largest number of IDEGs in HPC. This may indicate that immune dysregulation has a greater impact on the HPC. Therefore, the subsequent analysis will focus on four IDEGs in HPC.

#### 3.4.2 Construction of the PPI network and analysis of the relationship among IDEGs and other DEGs

The PPI networks were constructed for DEGs of HPC, *via* the STRING online database, to more clearly display the mutual interactions of the protein coded by DEGs. In HPC, the PPI network containing 144 nodes and 515 edges was built and visualized *via* Cytoscape software (**Figure 5B**). The average degree of NPY was 10, the average degree of OXTR was 122, the average degree of BLNK was 11, and the average degree of FGF12 was 11.

#### 3.4.3 Pathways analysis of IDEGs

Subsequently, the functional pathways analysis was performed about IDEGs and interacting genes in HPC (**Figure 5C**). In HPC, IDEGs and interacting genes are enriched in functional pathways, including the PI3K-Akt signaling pathway, focal adhesion, regulation of actin cytoskeleton, and neuroactive ligand-receptor interaction.

#### 3.4.4 ROC analysis

To investigate the diagnostic capacity of IDEG in SZ, the result of ROC analysis showed that areas under ROC curves (AUC) of *NPY*, *BLNK*, *OXTR*, and *FGF12* from the HPC were 0.87, 0.73, 0.85, and 0.89, respectively (**Figure 5D**); By logistic regression analysis, the complex result of *NPY*, *BLNK*, *OXTR*, and *FGF12* showed that the AUC was 0.95 (**Figure 5E**). These results revealed that these IDEGs might have a good application value and prospects for diagnosing clinical SZ.

### 3.5 Validation of IDEGs in the animal model

**Figure 6:**
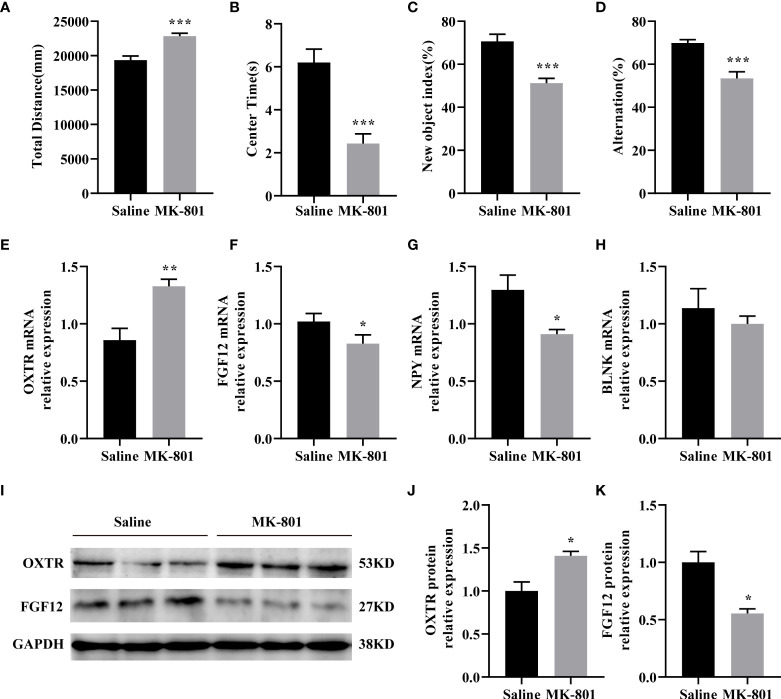
IDEGs are dysregulated in HPC of animal models of schizophrenia. **(A-D)** Behavioral experiments. **(A)** Significantly increased locomotor activity was recorded in the rat in the MK-801 group compared with that in the saline group (P < 0.001). **(B)** Amount of time mice spent in the center zone was significantly reduced in the MK-801 group compared with that in the saline group (P < 0.001). **(C)** The new object index of the MK-801 group was significantly lower than that of the saline group (P < 0.001). **(D)** Percentage of correct spontaneous alternation, with a significant decrease in the MK-801 group compared with the saline group (P < 0.001). **(E-H)** RT-qPCR analysis. **(E)** The mRNA expression levels of FGF12 (P < 0.05). **(F)** The mRNA expression levels of NPY (P < 0.05). **(G)** The mRNA expression levels of OXTR (P < 0.01). **(H)** The mRNA expression levels of BLNK (P > 0.05). **(I–K)** Western blotting analysis. **(I)** Representative immunoblots showing the protein levels of OXTR and FGF12 in the hippocampus were detected by Western blotting analysis. **(J)** The protein expression levels of OXTR (P < 0.05). **(K)** The protein expression levels of FGF12 (P < 0.05). Data are presented as Mean ± SEM. *P < 0.05, **P < 0.01, ***P < 0.001; unpaired two-tailed Student’s t-test. IDEGs, immune-related differentially expressed genes; HPC, hippocampus.

We established the MK-801 animal model of SZ and conducted behavioral experiments on the model. In the open field test, the total distance traveled by the rat that received repeated treatments with 0.5 mg/kg of MK-801 was significantly increased compared to that in the saline group (*t*_(14)_= 4.711, *P*<  0.001, **Figure 6A**), and spent less time in the central area (*t*_(14)_= 4.904, *P*< 0.001, **Figure 6B**). During the novel object recognition test, the New object index of the MK-801 group was significantly lower than that of the saline group(*t*_(14)_= 4.895, *P*< 0.001, **Figure 6C**). In the Y-maze test, Spontaneous alterations in arm entries were significantly lower in rats in the MK-801 group compared with the saline group (*t*_(14)_= 4.192, *P*< 0.001, **Figure 6D**). Thus, our modeling is successful from a behavioral perspective.

Next, we verified the results of the preliminary screening. In the HPC of rats, the mRNA levels of OXTR (*t*_(8)_= 3.919, *P*< 0.01) of the MK-801 group was significantly increased than that of the saline group (**Figure 6E**) the mRNA levels of FGF12 (*t*_(8)_ = 2.339, *P*< 0.05), NPY (*t*_(8)_ = 2.843, *P*<0.05) of the MK-801 group was significantly lower than that of the saline group (**Figures 6F–G**). while no difference was observed on the BLNK mRNA (*t*_(8)_= 0.7392, *P*> 0.05) level compared to the saline group (**Figure 6H**). Subsequent Western blotting results revealed reduced OXTR(*t*_(4)_ = 3.464, *P*< 0.05) and FGF12(*t*_(4)_ = 4.329, *P*< 0.05) protein expression levels (**Figures 6I–K**), almost consistent with previous HPC screening. Moreover, IDEGs from PFC and STR were also validated, but no significant difference was found.

## 4 Discussion

Mounting evidence shows that the immune system, especially immune cells and immune-related signaling molecules, is involved in the occurrence and progression of SZ (37). Several postmortem brains of schizophrenia studies have shown a reduction in synapse density (38), impaired neurodevelopment (39), and increased densities of T and B lymphocytes (40). Among the recruitment of immune cells, T cells show the ability to disrupt the blood-brain barrier (15), to infiltrate the brain, acting on microglia. And microglia are activated from a resting state to a pro-inflammatory state, releasing inflammatory mediators, excessive synaptic pruning, and aggravating oxidative stress damage (41). Hence, abnormal immune cells could directly or indirectly act on neurons, resulting in impaired brain function and aggravating clinical symptoms. We performed a comparative analysis of three brain regions to better understand the relationship between immune dysregulation and SZ. The analysis showed that in HPC, 100 DEGs and 42 pathways were associated with the immune system and there were 11 of 100 DEGs associated with Tem, in which *NPY*, *BLNK*, *OXTR*, and *FGF12* were IDEGs. In the PFC, there were 17 DEGs and 31 pathways related to the immune system and there were 10 of 17 DEGs associated with T-helper 1 cells, in which SERPINA3 and S100A9 were IDEGs. In the STR, 55 DEGs and 30 pathways were related to the immune system and there were 5 DEGs associated with T-helper 2 cells and 2 DEGs associated with mucosal-associated invariant T cells, in which S100A8 was IDEGs. It can be found that there are differences in the immune system in the three brain regions of SZ according to the above analysis result.

The current study found that T-cell subtypes with significant differences were different in three brain regions of patients with SZ compared to the control group. This finding is consistent with the aberrant aspects of T cells in SZ (41). Previous studies about gene expression profiling of postmortem brain tissue showed that the expression of immune/inflammatory-related genes increased and pointed out that abnormal immune/inflammatory response in the HPC may be the basis of the pathophysiology of SZ (42). Our study found abnormal Tem cells and four IDEGs (*NPY*, *BLNK*, *OXTR*, and *FGF12*) in HPC. At present, there is insufficient evidence to prove Tem number reduction in the brain tissue of SZ. But, the previous study on Tem and SZ found decreased CD4+ Tem cells in peripheral blood (43). Therefore, further work could investigate a change of Tem cells number in HPC and whether the peripheral blood Tem cell numbers changes can explain these changes in the brain tissue.

Afterward, correlation analyses were performed to confirm four IDEGs, in which there are three genes (*NPY*, *OXTR*, and *FGF12*) related to SZ supported by previous studies (26, 44). Such a result also clearly demonstrates the reliability of this study method. Neuropeptide Y, encoded by *NPY*, is widely distributed in the CNS and has multiple effects, mainly showing NPY participants in nerve and immune regulation. In terms of a postmortem autopsy study, the concentration of neuropeptides in the cerebral cortex of SZ patients is decreased (45, 46). The second IDEG identified in our research was *OXTR*. Oxytocin receptors (OXTR), the receptor for the hormone and neurotransmitter oxytocin (47), are the foundation of oxytocin to exert the formation and maintenance of social cognition, as well as social relationships function, and mainly exist in the limbic system and the reward system-related brain regions (44). In animal studies, OXT knockout mice showed SZ-like symptoms, while the expression of the OXTR in HPC was increased (48). When oxytocin receptors are antagonized, social fear of mice is increased (49). And studies also found that the polymorphism variation of the *OXTR* is related to nerve development disorders such as SZ (44). Furthermore, there have been no relevant studies about the relationship between SZ and *FGF12*, *BLNK.*

The functional analysis suggested that these genes may regulate the immune through the PI3K-Akt pathway and neural ligand-receptor interactions. The PI3K-Akt pathway is involved in regulating inflammation and metabolism-related signaling pathways to regulate the development, stability, and function of T cells (50). Postmortem autopsy, laboratory animal, and genetic studies have shown that the Akt signaling pathway is disturbed in SZ (51–55).

Also, these genes have high diagnostic value and important clinical significance. Therefore, future studies can analyze the relationship between the central and peripheral gene expression levels to better apply them to clinical practice from the perspective of immunity. Analysis relationship among SZ and differential immune cells, IRG, and functional pathways in HPC have significant implications. Therefore, the decrease in the number of Tem cells in the HPC of SZ may be regulated by the PI3K/AKT signaling pathway and neural ligand-receptor interactions.

And analysis results also suggested that NPY and OXTR indirectly regulate the immune system by neuroactive ligand-binding receptors interaction. The probable interpretation is that the genes can interact with neurotransmitters related to mental diseases, such as dopamine (56), and there is cross-talk between neurotransmitters and immune cells (57). On the one hand, dopamine and neuropeptides could bind to dopamine receptors expressed on T cells, and directly activate or inhibit the T-cell function (58–61). On the other hand, neurotransmitters and neuropeptides could increase gene expression, cytokine secretion, chemotaxis, immune cell apoptosis, cell proliferation, and so on (62).

Our study found that the IDEGs screened in the HPC were consistent with the expression trend (except BLNK) in SZ animal models. These results also were consistent with previous studies that found Anti-N-methyl-D-aspartate receptor (NMDAR) hypofunction related to inflammation (63). These findings indicated that the increase of NMDAR activity may cause the downregulation of the oxytocin signaling pathway (64) and that MK-801 can significantly decrease NPY immune reactivity (65). In addition, there is a lack of reports about the relationship between BLNK and FGF12 and NMDAR, among which FGF12 is associated with voltage-gated sodium channels (66), and NMDAR that are highly permeable to Ca^2+^ can regulate voltage-gated sodium channels (67). The previous study found that NMDAR can regulate FGF12, suggesting that NMDAR can regulate voltage-gated sodium channels by regulating FGF12. BLNK plays a vital role during B cells development (68). A large body of literature has reported that B cells are abnormal in SZ (69) but decreased BLNK expression was not found in this animal model of SZ, which deserves further investigation.

Besides the above-mentioned immune cells, other immune cells should be mentioned, such as microglia. As macrophages of the CNS, microglia haven’t significant differences in the abundance of cells in our study. Still, we found that the expression of *P2RY12*, a marker of microglial function shift, is down-regulated (70), which also indicates the activation of microglia in SZ.

Inevitably, there are some limitations in this study. First, expression profiles of genes could be affected by confounding factors, such as individual variability among samples, small sample size, and platforms. Therefore, adding more chip data and rigorous quality control is needed to minimize the errors. Second, different immune infiltration analysis tools have different algorithms, and the types of immune cells analyzed are also different. So, we tried to add other immune infiltration analysis algorithms, such as the Xcell algorithm, as compliments and validation of the ImmuCellAI algorithm. And single-cell sequencing is an effective strategy in our future studies to clarify the interaction among immune cells. Third, because of the difficulty of obtaining autopsy brain tissue samples, a rodent disease animal model for validation was adopted, adding different datasets, and using different algorithms to analyze. To better verify our findings and conduct in-depth research, we will try to perform multi-angle verification in disease models with various modeling schemes. In addition, next-generation sequencing of rat brain tissue and using algorithms to estimate immune cell abundance is also an effective strategy. Our results provide a new perspective for an in-depth analysis of the mechanism research of SZ.

**Figure 7:**
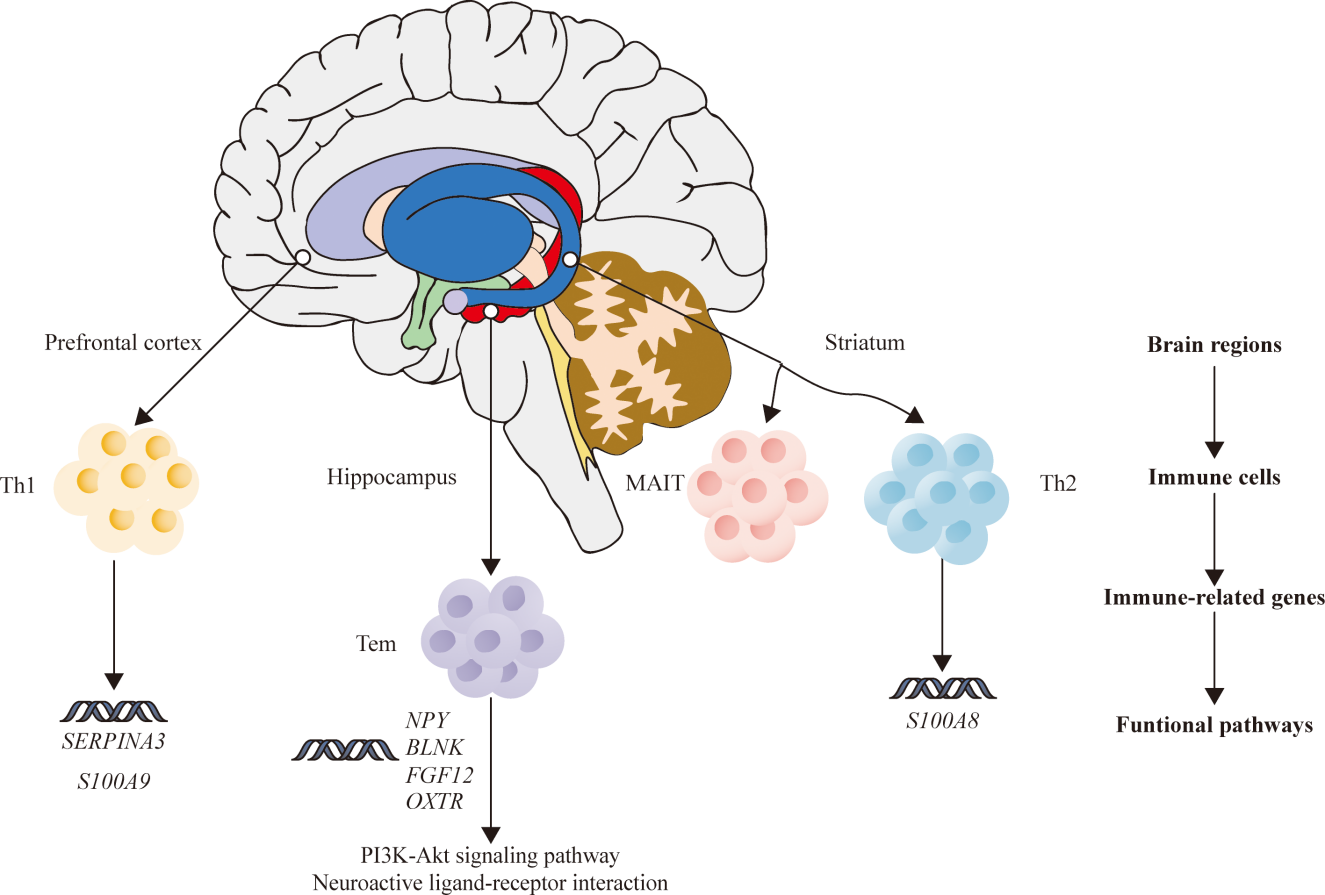
The relationship among brain regions, immune cells, immune-related DEGs, and functional pathways of schizophrenia. DEGs, differentially expressed genes.

In summary, the current study analyzed the relationship between immune disorders and SZ by horizontally comparing the DEGs, infiltrating immune cells, and IDEGs in three brain areas. Our results inferred that HPC in SZ may be more closely related to immune disorders compared with PFC and STR. Meanwhile, this study proposes a hypothesis that the abnormal expression of four genes in HPC of SZ may indirectly affect Tem cells by acting on the PI3K-Akt signal pathway and neuroactive ligand-binding receptors interaction, according to the analysis results (**Figure 7**).

## Data availability statement

The datasets presented in this study can be found in online repositories. The names of the repository/repositories and accession number(s) can be found in the article/**Supplementary Material**.

## Ethics statement

All animal procedures were approved by the Institutional Animal Care and Use Committee of Shanxi Medical University

## Author contributions

YX, SL, YG, and YD provided the concept and designed the study. GW, ZL, XD, JL, XL conducted the analyses and wrote the manuscript. YG, YD, and GW participated in data analysis. SL, YG, GW, and ZL contributed to revising and proofreading the manuscript. All authors contributed to the article and approved the submitted version.

## Funding

We also are grateful for the support from the National Natural Science Foundation of China (81971601, 81701326); Shanxi Provincial Science and technology achievements transformation and guidance project (201904D131020); Shanxi Provincial Department of Education University Science and technology innovation plan project (202010203); Shanxi Provincial Science and technology innovation team of multidisciplinary diagnosis and treatment of cognitive impairment (201705D131027).

## Acknowledgments

We appreciate Lanz TA et al. who submitted the chip’s data to the GEO databases.

## Conflict of Interest

The authors declare that the research was conducted in the absence of any commercial or financial relationships that could be construed as a potential conflict of interest.

## Publisher’s note

All claims expressed in this article are solely those of the authors and do not necessarily represent those of their affiliated organizations, or those of the publisher, the editors and the reviewers. Any product that may be evaluated in this article, or claim that may be made by its manufacturer, is not guaranteed or endorsed by the publisher.

## Footnotes

1
http://sangerbox.com/Tool.

2
http://www.webgestalt.org/.

3
http://bioinfo.life.hust.edu.cn/web/ImmuCellAI/.

4
https://xcell.ucsf.edu.

5
https://www.immport.org/.

6
http://string-db.org/.

7
https://david.ncifcrf.gov.

8
https://www.omicstudio.cn/tool.
