# Supplementary figures and images for: Exploration of the relationship between hippocampus and immune system in schizophrenia based on immune infiltration analysis
Source: Front Immunol. 2022 Aug 2;13:878997. doi: 10.3389/fimmu.2022.878997 (PMC9380889; doi:10.3389/fimmu.2022.878997)

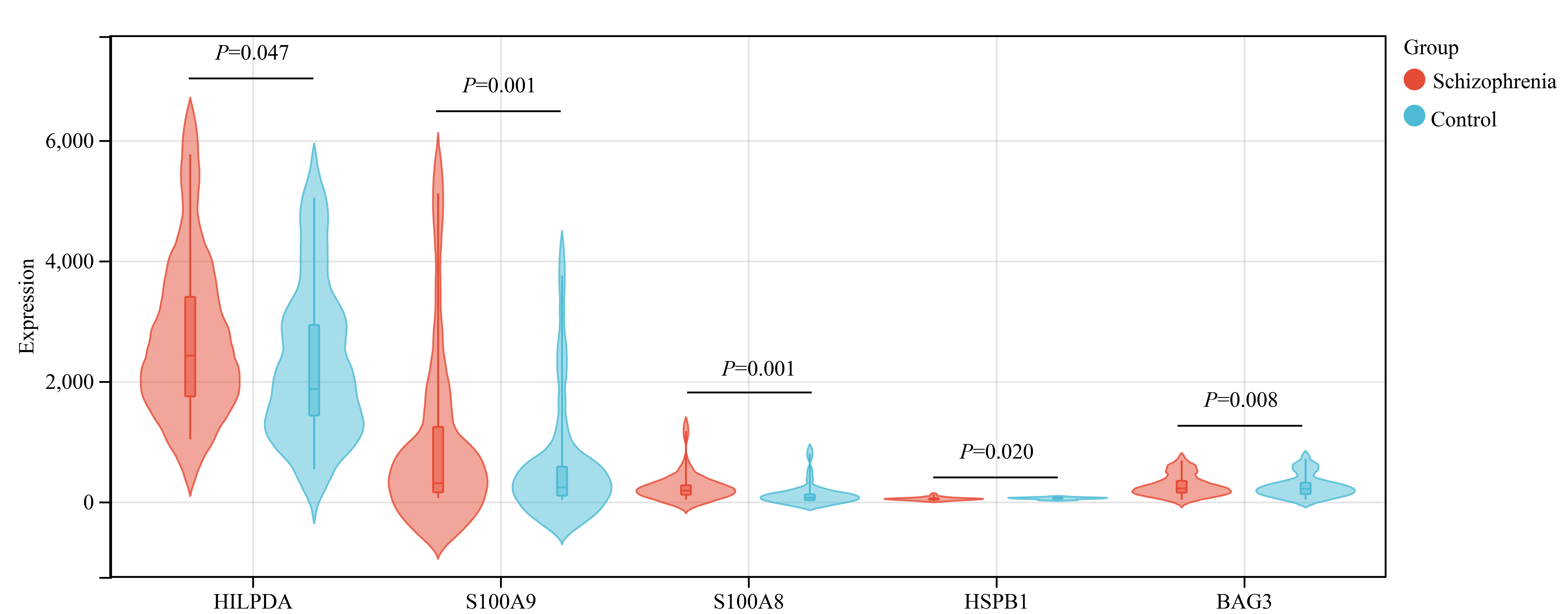

Supplement: Supplementary file 1 [file Image_1.tif]

(A)

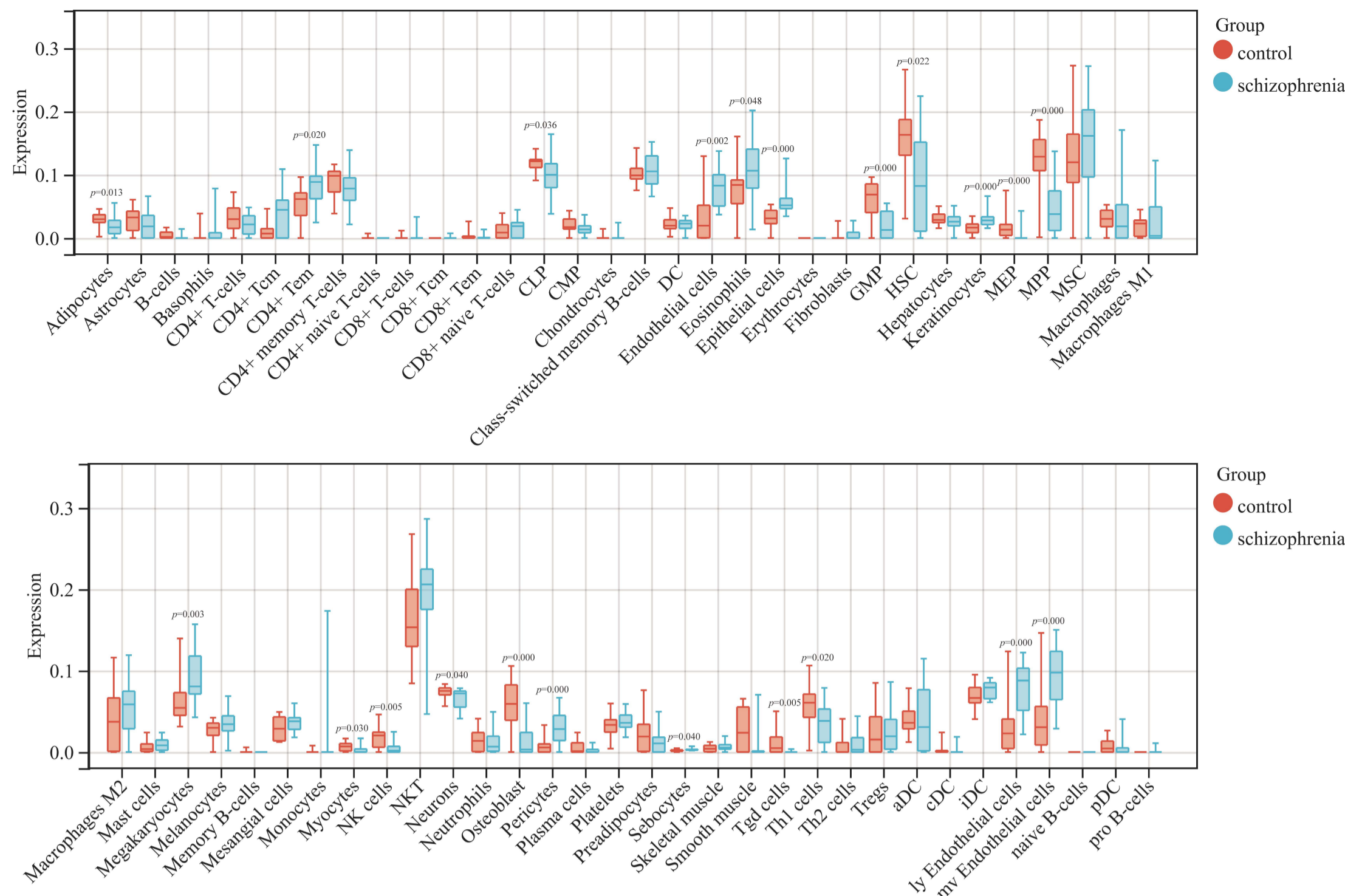

(B)

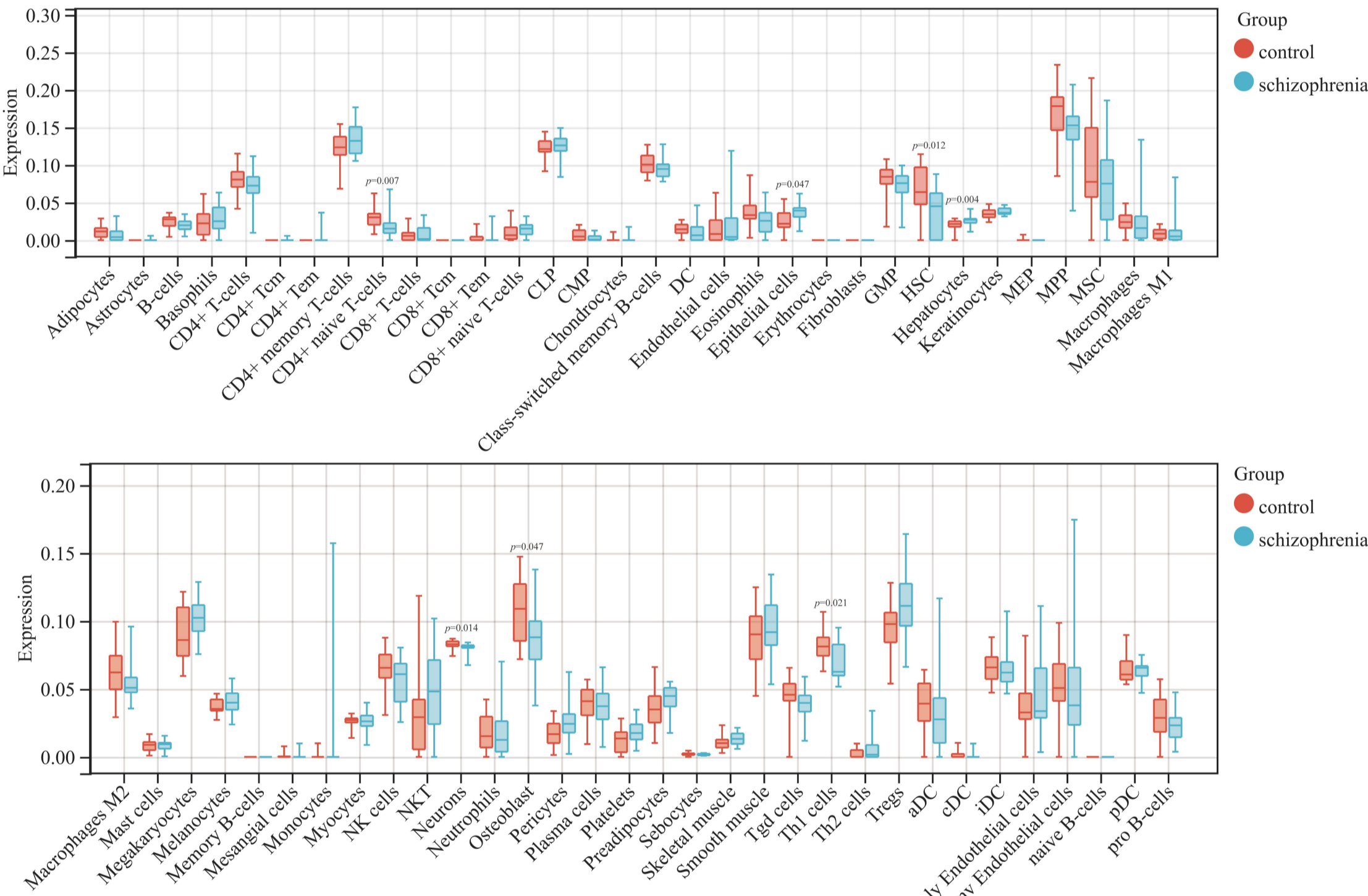

(C)

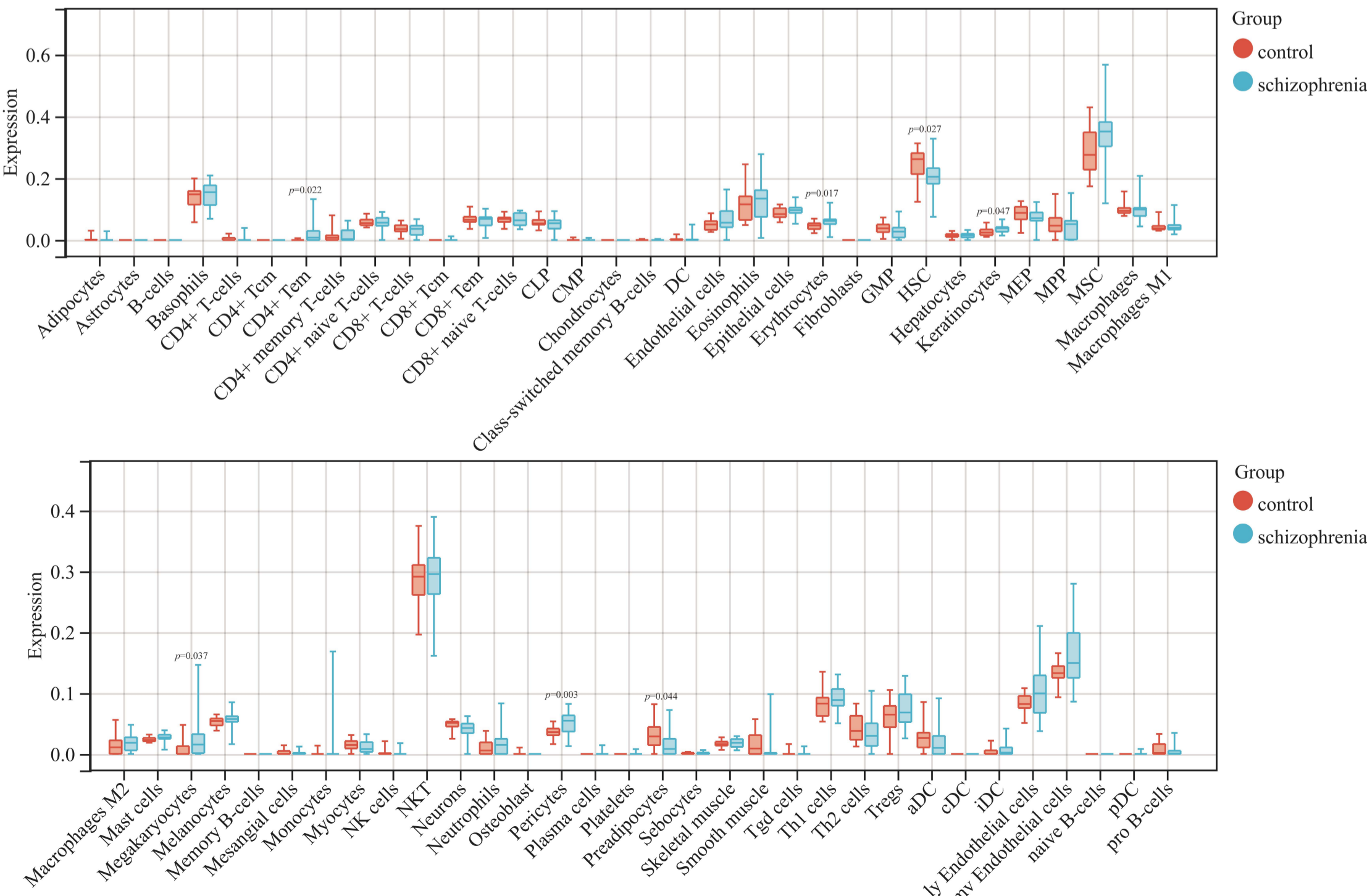

Supplement: Supplementary file 2 [file Image_2.pdf]

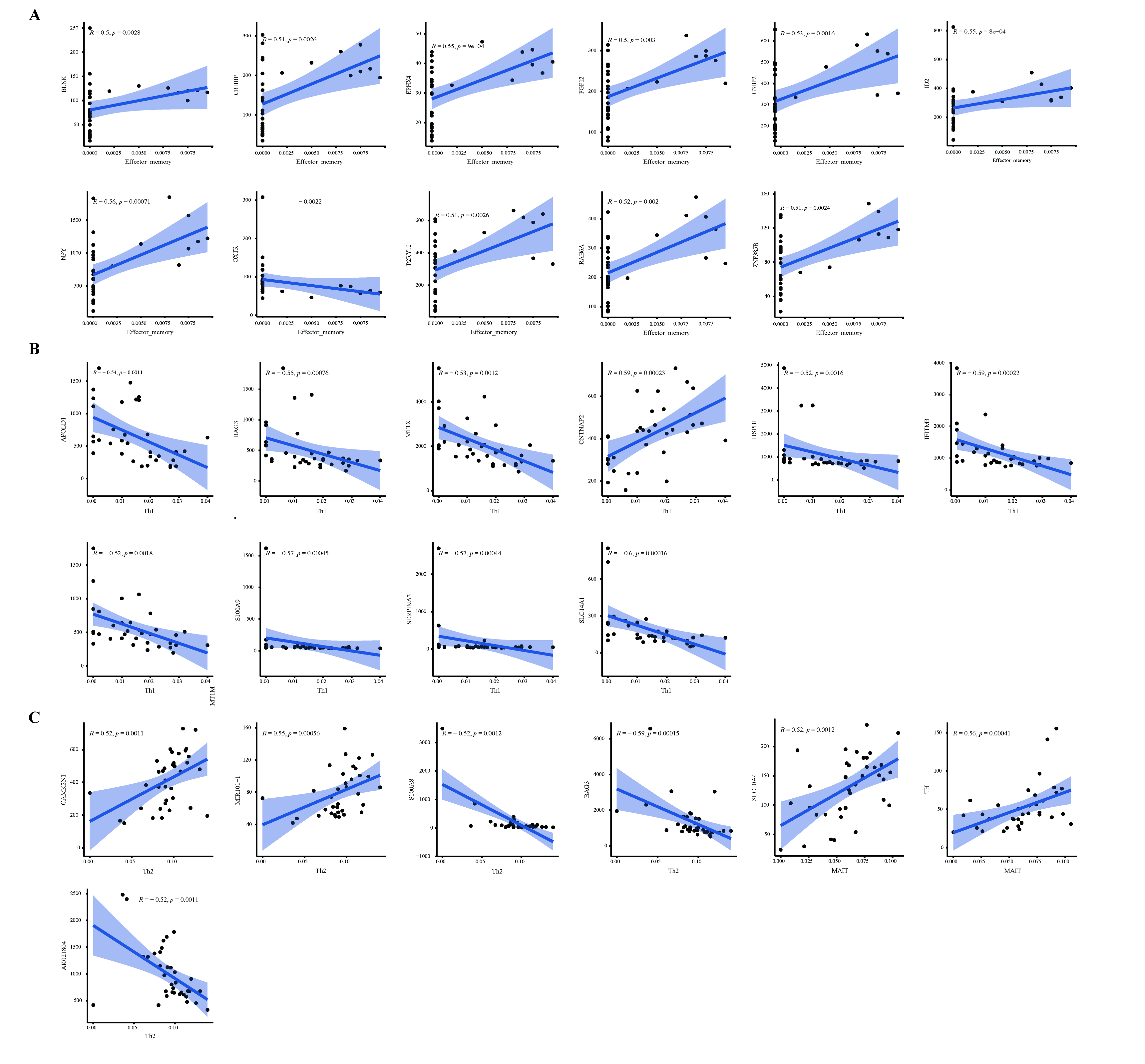

Supplement: Supplementary file 4 [file Image_4.tif]
