# Supplementary material for: Exploration of the relationship between hippocampus and immune system in schizophrenia based on immune infiltration analysis
Source: Front Immunol. 2022 Aug 2;13:878997. doi: 10.3389/fimmu.2022.878997 (PMC9380889; doi:10.3389/fimmu.2022.878997)
Supplement: Supplementary file 3 [file Image_3.pdf]

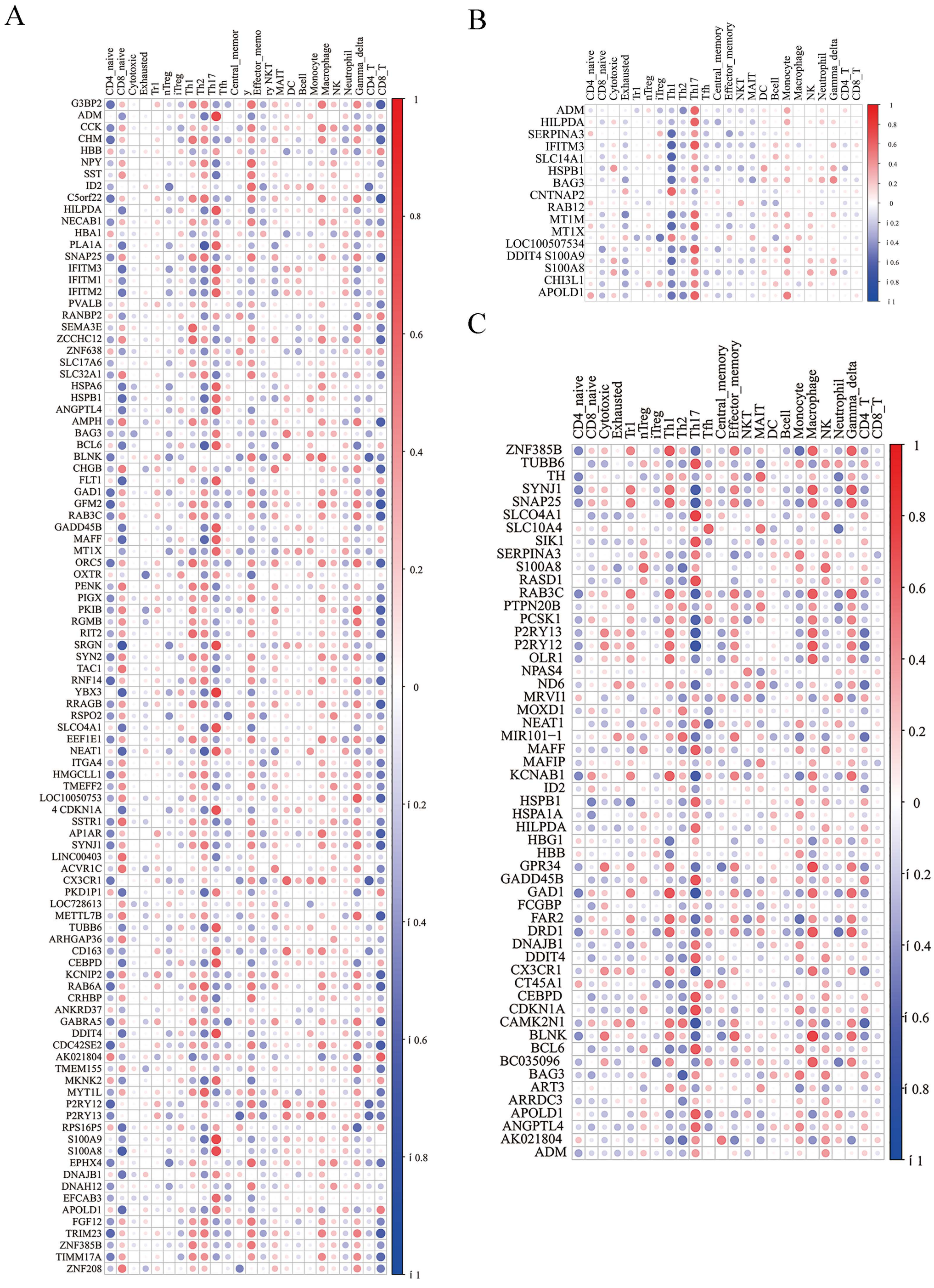
A
CD4_naive
CD8_naive
Cytotoxic
Exhausted
Tr1
nTreg
iTreg
Th1
Th2
Th17
Tfh
Central_memor
y
Effector_memo
ry NKT
MAIT
DC
Bcell
Monocyte
Macrophage
NK
Neutrophil
Gamma_delta
CD4_T
CD8_T
G3BP2
ADM
CCK
CHM
HBB
NPY
SST
ID2
C5orf22
HILPDA
NECAB1
HBA1
PLA1A
SNAP25
IFITM3
IFITM1
IFITM2
PVALB
RANBP2
SEMA3E
ZCCHC12
ZNF638
SLC17A6
SLC32A1
HSPA6
HSPB1
ANGPTL4
AMPH
BAG3
BCL6
BLNK
CHGB
FLT1
GAD1
GFM2
RAB3C
GADD45B
MAFF
MT1X
ORC5
OXTR
PENK
PIGX
PKIB
RGMB
RIT2
SRGN
SYN2
TAC1
RNF14
YBX3
RRAGB
RSPO2
SLCO4A1
EEF1E1
NEAT1
ITGA4
HMGCLL1
TMEFF2
LOC10050753
4 CDKN1A
SSTR1
AP1AR
SYNJ1
LINC00403
ACVR1C
CX3CR1
PKD1P1
LOC728613
METTL7B
TUBB6
ARHGAP36
CD163
CEBPD
KCNIP2
RAB6A
CRHBP
ANKRD37
GABRA5
DDIT4
CDC42SE2
AK021804
TMEM155
MKNK2
MYT1L
P2RY12
P2RY13
RPS16P5
S100A9
S100A8
EPHX4
DNAJB1
DNAH12
EFCAB3
APOLD1
FGF12
TRIM23
ZNF385B
TIMM17A
ZNF208
1
0.8
0.6
0.4
0.2
0
í 0.2
í 0.4
í 0.6
í 0.8
í 1
B
CD4_naive
CD8_naive
Cytotoxic
Exhausted
Tr1
nTreg
iTreg
Th1
Th2
Th17
Tfh
Central_memory
Effector_memory
NKT
MAIT
DC
Bcell
Monocyte
Macrophage
NK
Neutrophil
Gamma_delta
CD4_T
CD8_T
ADM
HILPDA
SERPINA3
IFITM3
SLC14A1
HSPB1
BAG3
CNTNAP2
RAB12
MT1M
MT1X
LOC100507534
DDIT4 S100A9
S100A8
CHI3L1
APOLD1
1
0.8
0.6
0.4
0.2
0
í 0.2
í 0.4
í 0.6
í 0.8
í 1
C
CD4_naive
CD8_naive
Cytotoxic
Exhausted
Tr1
nTreg
iTreg
Th1
Th2
Th17
Tfh
Central_memory
Effector_memory
NKT
MAIT
DC
Bcell
Monocyte
Macrophage
NK
Neutrophil
Gamma_delta
CD4_T
CD8_T
ZNF385B
TUBB6
TH
SYNJ1
SNAP25
SLCO4A1
SLC10A4
SIK1
SERPINA3
S100A8
RASD1
RAB3C
PTPN20B
PCSK1
P2RY13
P2RY12
OLR1
NPAS4
ND6
MRVI1
MOXD1
NEAT1
MIR101−1
MAFF
MAFIP
KCNAB1
ID2
HSPB1
HSPA1A
HILPDA
HBG1
HBB
GPR34
GADD45B
GAD1
FCGBP
FAR2
DRD1
DNAJB1
DDIT4
CX3CR1
CT45A1
CEBPD
CDKN1A
CAMK2N1
BLNK
BCL6
BC035096
BAG3
ART3
ARRDC3
APOLD1
ANGPTL4
AK021804
ADM
1
0.8
0.6
0.4
0.2
0
í 0.2
í 0.4
í 0.6
í 0.8
í 1
