# Supplementary material for: Exploration of the relationship between hippocampus and immune system in schizophrenia based on immune infiltration analysis
Source: Front Immunol. 2022 Aug 2;13:878997. doi: 10.3389/fimmu.2022.878997 (PMC9380889; doi:10.3389/fimmu.2022.878997)
Supplement: Supplementary file 5 [file DataSheet_1.docx]

Supplementary Material

# Supplemental Methods

## Open Field Test

After rats received MK-801 for 14 consecutive days, they were placed in the center of a square open field (50 × 50 × 50 cm) and allowed to move freely for 5 min. After testing each rat, the test apparatus was cleaned with 75% alcohol solution and alcohol was volatilized to clean before testing the next rat. During the experiment, the time that each rat spent in the middle of the open field and the total distance moved were recorded using an automatic video tracking system (TMV-100S, TECHMAN, Chengdu, China).

## Novel Object Recognition Test

The novel object recognition test was conducted in an open field apparatus (60 × 60 × 50 cm). The experiment was divided into two periods: familiarity period and test period. During the familiarity period, the rats were recorded to explore two identical objects freely in the open field within 10 minutes. After six hours, one of the objects was replaced with a completely different new object and placed in the same position. The rats spent 10 minutes exploring the old and new objects. We used the following formula to calculate the new object index (NOI): new object recognition index = time to explore new objects /(time to explore new objects + time to explore old objects) × 100%. The recording was done using a video tracking system (Ethovision 3.0, Noldus Information Technology, Wageningen, Netherlands).

## Y-Maze Test

The Y-maze was a three arm isocenter maze with each arm measuring 40cm in length, 17cm in height, and 15cm in width. Each rat was placed in the central triangular area, which was allowed to walk freely in the maze for 8 min, and the smart 3.0 software system was employed to record the order and number of times the rat entered the arm. A correct spontaneous alternation was identified as a set of three different arm entries. The percentage of spontaneous correct alternations was calculated as (number of correct alternations) / (number of total arms − 2) × 100%.

## Real-Time Quantitative Reverse transcription-polymerase Chain Reaction (RT-qPCR)

The hippocampus tissue was taken from all rats immediately after behavior tests and stored at −80°C. Total RNA was extracted from the HPC using Transzol Up Plus RNA Kit (TransGen Biotech, Beijing, China). RNA was quantitated using a NanoDrop (Thermo Fisher, USA). RNA integrity was checked by RNA electrophoresis. Messenger (m) RNA was converted into cDNA using a TransStart Uni All-in-One First-Strand cDNA Synthesis SuperMix for qPCR (One-Step-gDNA Removal) (TransGen Biotech, Beijing, China). The expression of specific mRNAs was assayed using fluorescence-based real-time quantitative PCR (RT-qPCR). Quantitative PCR reactions were performed using PerfectStart Green qPCR SuperMix (TransGen Biotech, Beijing, China)in triplicate for each sample. GAPDH was chosen as the reference gene. The amplification cycles were 94 C for 5 s and 60 C for 30 s. At the end of the assay, a melting curve was constructed to evaluate the specificity of the reaction. All quantitative real-time PCR reactions used a CFX Connect Real-Time PCR Detection System (Bio-Rad, USA). Differences in expression between treatment groups were determined by the 2^–ΔΔCt^ method. The primers used are shown in **Supplementary Table 2**.

## Western blotting

Total protein extraction used Proteinext Mammalian Total Protein Extraction Kit (TransGen Biotech, Beijing, China). The protein concentration was tested on the BCA kit (Boster, Wuhan, China). Protein along with the sample loading buffer was boiled before loading onto SDS-PAGE. Proteins were electrophoretically transferred onto nitrocellulose membrane, blocked with a blocking buffer (5% skim milk in 0.1% TBST). Following, membranes were incubated in primary antibody (in 0.1% TBST), antiFGF12 (1:500, Affinity Biosciences,USA), antiOXTR (1:500, Bioss, Beijing, China), and antiGAPDH (1:500, Bioss, Beijing, China). The next day after washes, membranes were incubated with the secondary antibody (1:500, TransGen Biotech, Beijing, China), immunoreactivity was detected with an ECL Western Blotting Detection Kit (Boster, Wuhan, China). The Protein bands were quantified using ImageJ.

## Statistical analysis

Behavioral testing, RT-qPCR and Western Blot were analyzed with an Unpaired two-tailed t-test. Normality and homogeneity of variance were verified using the Shapiro–Wilk test and Levene's test, respectively. Data are described by means ± SEMs. Non-normal distribution data were calculated using the Wilcoxon rank-sum test.

# Supplementary Figures and Tables

## Supplementary Figure

**Supplementary Figure 1| Violin plot of five gene expression levels in the prefrontal cortex between control and schizophrenia group.** The red and blue bars represent the schizophrenia and control groups, respectively.

**Supplementary Figure 2| Box plot of 64 cell types via xCell algorithm.** The red and blue bars represent the SZ and control groups, respectively. **(A)**HPC **(B)**PFC **(C)**STR. HPC, hippocampus; PFC, prefrontal cortex; STR, striatum.

**Supplementary Figure 3| The matrix heatmaps of the DEGs and infiltrating immune cells.**

The size of the colored circles represents the strength of the correlation; the red circles represent a positive correlation, and the blue circles represent a negative correlation. The darker the color, the higher the correlation. **(A)**HPC **(B)**PFC **(C)**STR. HPC, hippocampus; PFC, prefrontal cortex; STR, striatum.

**Supplementary Figure 4| The correlation plots between DEGs and differential immune cells.**

Analysis was performed *via* Spearman correlation. R, Spearman coefficient. The blue line represents the linear prediction. The blue belt is the 95% confidence interval; points are the value of DEGs in all sample values. |correlation coefficient| ≥ 0.5, *P*< 0.05 were considered statistically significant. **(A)**HPC **(B)**PFC **(C)**STR. HPC, hippocampus; PFC, prefrontal cortex; STR, striatum.

## Supplementary Tables

**Supplementary Table 1| Basic information of brain region samples used for** **the statistical analysis.**

|  | GSE53987 | | | | | | GSE17612 | |
| --- | --- | --- | --- | --- | --- | --- | --- | --- |
|  | Hippocampus | | Prefrontal cortex | | Striatum | | Prefrontal cortex | |
|  | SZ | Control | SZ | Control | SZ | Control | SZ | Control |
| Number  of sample | 15 | 18 | 15 | 19 | 18 | 18 | 28 | 23 |
| Gender (Females/males) | 6/9 | 9/9 | 8/7 | 9/10 | 8/10 | 8/10 | 9/19 | 10/13 |
| Average age | 45.73±2.2688 | 48.12±2.5798 | 46.00±2.2297 | 48.05±2.4429 | 45±2.0627 | 48.44±2.5491 | 73.32±2.8730 | 69.04±4.4944 |
| *P*-value | 0.4928 | | 0.5493 | | 0.3009 | | 0.4109 | |
| Postmortem interval (hours) | 19.40±1.8591 | 19.39±1.2263 | 18.91±1.7275 | 19.53±1.1681 | 19.90±1.6667 | 19.75±1.2109 | 8.61±1.3366 | 9.90±0.9152 |
| *P*-value | 0.9943 | | 0.7625 | | 0.9452 | | 0.0569 | |
| Brain pH | 6.43±0.0798 | 6.61±0.0504 | 6.52±0.1001 | 6.59±0.0504 | 6.47±0.0876 | 6.59±0.0533 | - | - |
| *P*-value | 0.5144 | | 0.5144 | | 0.2313 | | - | |

**Supplementary Table 2| Primer pairs used for RT-qPCR experiment**

| Gene target | Forward sequence (5′ to 3′) | Reverse sequence (5′ to 3′) |
| --- | --- | --- |
| FGF12 | TTCAGCCAGCAGGGATATTT | TCTCCATTCATGGCCACATA |
| NPY | TACTACTCCGCTCTGCGACA | AAGGGTCTTCAAGCCTTGTTCT |
| OXTR | TCGTACTGGCCTTCATCGTG | TGAAGGCAGAAGCTTCCTTGG |
| BLNK | TAACTGTCCCTGCCAGTCAGA | GAGGTCCTTTGACTTTTAGCTTCT |
| GAPDH | AGGGTGGTGGACCTCATGG | AGCAACTGAGGGCCTCTCTCTT |

**Supplementary Table 3| Differentially expressed genes** **in the hippocampus between schizophrenia and control groups**

| Gene names | log_2_FC | *P*-Value | Regulated |
| --- | --- | --- | --- |
| S100A8 | 1.646297 | 0.00134391 | Up-Regulated |
| APOLD1 | 1.323602 | 0.00012791 | Up-Regulated |
| ADM | 1.229347 | 0.00089754 | Up-Regulated |
| MAFF | 1.214094 | 0.00025011 | Up-Regulated |
| HILPDA | 1.203211 | 0.00049175 | Up-Regulated |
| DDIT4 | 1.131786 | 0.00000458 | Up-Regulated |
| SLCO4A1 | 0.926689 | 0.00061936 | Up-Regulated |
| HSPB1 | 0.925759 | 0.00114044 | Up-Regulated |
| AK021804 | 0.834809 | 0.00001781 | Up-Regulated |
| S100A9 | 0.818157 | 0.00991745 | Up-Regulated |
| IFITM1 | 0.800544 | 0.00016426 | Up-Regulated |
| IFITM2 | 0.800405 | 0.00009162 | Up-Regulated |
| BAG3 | 0.791611 | 0.00058924 | Up-Regulated |
| ANGPTL4 | 0.761735 | 0.00011009 | Up-Regulated |
| CEBPD | 0.755783 | 0.00008445 | Up-Regulated |
| EFCAB3 | 0.743974 | 0.00627877 | Up-Regulated |
| CD163 | 0.73824 | 0.03697287 | Up-Regulated |
| YBX3 | 0.734843 | 0.00057363 | Up-Regulated |
| IFITM3 | 0.727421 | 0.00014884 | Up-Regulated |
| PLA1A | 0.709418 | 0.00424859 | Up-Regulated |
| CDKN1A | 0.700667 | 0.00131080 | Up-Regulated |
| BCL6 | 0.692834 | 0.00078502 | Up-Regulated |
| PKD1P1 | 0.690609 | 0.00010574 | Up-Regulated |
| HBB | 0.67939 | 0.02523251 | Up-Regulated |
| ANKRD37 | 0.671983 | 0.00387062 | Up-Regulated |
| RANBP2 | 0.669503 | 0.00072059 | Up-Regulated |
| HBA1 | 0.66659 | 0.02647912 | Up-Regulated |
| HSPA6 | 0.660209 | 0.02892088 | Up-Regulated |
| MT1X | 0.650778 | 0.00085977 | Up-Regulated |
| MIR612 | 0.638193 | 0.00435647 | Up-Regulated |
| TUBB6 | 0.637233 | 0.00494861 | Up-Regulated |
| RPS16P5 | 0.625177 | 0.00061834 | Up-Regulated |
| GADD45B | 0.624805 | 0.00293393 | Up-Regulated |
| ZNF638 | 0.615199 | 0.00002626 | Up-Regulated |
| SRGN | 0.607839 | 0.03745333 | Up-Regulated |
| FLT1 | 0.606228 | 0.00024874 | Up-Regulated |
| MKNK2 | 0.591931 | 0.00002629 | Up-Regulated |
| OXTR | 0.588161 | 0.00032770 | Up-Regulated |
| DNAJB1 | 0.585091 | 0.02487615 | Up-Regulated |
| DNAH12 | -0.59388 | 0.00020511 | Down-Regulated |
| ACVR1C | -0.59564 | 0.00033766 | Down-Regulated |
| RRAGB | -0.59623 | 0.00082912 | Down-Regulated |
| PIGX | -0.59646 | 0.00002418 | Down-Regulated |
| SEMA3E | -0.59684 | 0.00050049 | Down-Regulated |
| METTL7B | -0.59701 | 0.00228921 | Down-Regulated |
| LINC00403 | -0.59826 | 0.00144107 | Down-Regulated |
| MYT1L | -0.60142 | 0.00533090 | Down-Regulated |
| CDC42SE2 | -0.60278 | 0.00006731 | Down-Regulated |
| EPHX4 | -0.60611 | 0.00011669 | Down-Regulated |
| AMPH | -0.60842 | 0.00003489 | Down-Regulated |
| AP1AR | -0.60845 | 0.00005741 | Down-Regulated |
| PENK | -0.61415 | 0.00059867 | Down-Regulated |
| ITGA4 | -0.61478 | 0.00032031 | Down-Regulated |
| RIT2 | -0.61519 | 0.00019580 | Down-Regulated |
| ORC5 | -0.61703 | 0.00009536 | Down-Regulated |
| GABRA5 | -0.62071 | 0.00797666 | Down-Regulated |
| ZNF208 | -0.62293 | 0.00493954 | Down-Regulated |
| HMGCLL1 | -0.63127 | 0.00001163 | Down-Regulated |
| ZCCHC12 | -0.63958 | 0.00144142 | Down-Regulated |
| RGMB | -0.64946 | 0.00007388 | Down-Regulated |
| TMEFF2 | -0.6558 | 0.00005424 | Down-Regulated |
| ID2 | -0.65971 | 0.00618089 | Down-Regulated |
| CHGB | -0.66053 | 0.00067224 | Down-Regulated |
| C5orf22 | -0.66936 | 0.00077790 | Down-Regulated |
| SSTR1 | -0.67552 | 0.00039078 | Down-Regulated |
| EEF1E1 | -0.68468 | 0.00000612 | Down-Regulated |
| FGF12 | -0.69289 | 0.00001888 | Down-Regulated |
| RAB6A | -0.69369 | 0.00068116 | Down-Regulated |
| TRIM23 | -0.7009 | 0.00011849 | Down-Regulated |
| KCNIP2 | -0.70331 | 0.00115883 | Down-Regulated |
| ZNF385B | -0.70809 | 0.00065820 | Down-Regulated |
| GAD1 | -0.71088 | 0.00019009 | Down-Regulated |
| GFM2 | -0.72446 | 0.00017887 | Down-Regulated |
| TMEM155 | -0.72688 | 0.00296064 | Down-Regulated |
| CHM | -0.73763 | 0.00036806 | Down-Regulated |
| TIMM17A | -0.7431 | 0.00005223 | Down-Regulated |
| G3BP2 | -0.7546 | 0.00009329 | Down-Regulated |
| RNF14 | -0.75501 | 0.00002916 | Down-Regulated |
| P2RY13 | -0.77507 | 0.00590487 | Down-Regulated |
| SNAP25 | -0.77605 | 0.00021071 | Down-Regulated |
| LOC728613 | -0.77918 | 0.01319887 | Down-Regulated |
| BLNK | -0.78494 | 0.00634307 | Down-Regulated |
| NECAB1 | -0.79928 | 0.00002666 | Down-Regulated |
| RSPO2 | -0.81927 | 0.00148193 | Down-Regulated |
| PKIB | -0.88147 | 0.00230647 | Down-Regulated |
| TAC1 | -0.89378 | 0.00067768 | Down-Regulated |
| SYN2 | -0.90257 | 0.00000833 | Down-Regulated |
| CCK | -0.90886 | 0.00002074 | Down-Regulated |
| SLC17A6 | -0.91789 | 0.01063887 | Down-Regulated |
| SYNJ1 | -0.92037 | 0.00003537 | Down-Regulated |
| LOC100507534 | -0.93971 | 0.00129654 | Down-Regulated |
| SLC32A1 | -0.94625 | 0.00000175 | Down-Regulated |
| RAB3C | -0.97321 | 0.00006732 | Down-Regulated |
| ARHGAP36 | -1.07131 | 0.00178527 | Down-Regulated |
| CX3CR1 | -1.12223 | 0.00169403 | Down-Regulated |
| NPY | -1.14085 | 0.00006005 | Down-Regulated |
| PVALB | -1.16851 | 0.00037690 | Down-Regulated |
| SST | -1.18617 | 0.00002084 | Down-Regulated |
| P2RY12 | -1.25307 | 0.00066415 | Down-Regulated |
| CRHBP | -1.26291 | 0.00000112 | Down-Regulated |

**Supplementary Table 4| Differentially expressed genes in the prefrontal cortex between schizophrenia and control groups**

| Gene names | log_2_FC | *P*-Value | Regulated |
| --- | --- | --- | --- |
| S100A8 | 1.18930 | 0.01057212 | Up-Regulated |
| SERPINA3 | 1.02520 | 0.00808683 | Up-Regulated |
| BAG3 | 1.02373 | 0.00001089 | Up-Regulated |
| APOLD1 | 0.86771 | 0.00413971 | Up-Regulated |
| ADM | 0.86351 | 0.01077194 | Up-Regulated |
| DDIT4 | 0.80529 | 0.00142993 | Up-Regulated |
| MT1M | 0.68507 | 0.00149654 | Up-Regulated |
| S100A9 | 0.62639 | 0.03768784 | Up-Regulated |
| MT1X | 0.62526 | 0.00099529 | Up-Regulated |
| IFITM3 | 0.62195 | 0.00008875 | Up-Regulated |
| HILPDA | 0.62051 | 0.01107753 | Up-Regulated |
| CHI3L1 | 0.61169 | 0.00329246 | Up-Regulated |
| SLC14A1 | 0.61108 | 0.02459944 | Up-Regulated |
| HSPB1 | 0.60668 | 0.00475464 | Up-Regulated |
| CNTNAP2 | -0.60920 | 0.00018782 | Down-Regulated |
| RAB12 | -0.60960 | 0.01160787 | Down-Regulated |
| LOC100507534 | -0.67670 | 0.00000286 | Down-Regulated |

**Supplementary Table 5| Differentially expressed genes in the striatum between schizophrenia and control groups**

| Gene names | log_2_FC | *P*-Value | Regulated |
| --- | --- | --- | --- |
| CT45A1 | 1.16457 | 0.00767603 | Up-Regulated |
| S100A8 | 1.13954 | 0.01625809 | Up-Regulated |
| SERPINA3 | 1.07912 | 0.02050487 | Up-Regulated |
| MAFF | 1.06724 | 0.00507322 | Up-Regulated |
| APOLD1 | 0.96352 | 0.00198518 | Up-Regulated |
| CEBPD | 0.93324 | 0.00206726 | Up-Regulated |
| DDIT4 | 0.84379 | 0.00213820 | Up-Regulated |
| ADM | 0.84261 | 0.01253146 | Up-Regulated |
| BAG3 | 0.82791 | 0.00025715 | Up-Regulated |
| RASD1 | 0.80828 | 0.00000474 | Up-Regulated |
| HILPDA | 0.79832 | 0.00997632 | Up-Regulated |
| AK021804 | 0.79167 | 0.00023706 | Up-Regulated |
| BCL6 | 0.78198 | 0.00408499 | Up-Regulated |
| GADD45B | 0.77756 | 0.00023493 | Up-Regulated |
| DNAJB1 | 0.74831 | 0.00832588 | Up-Regulated |
| ANGPTL4 | 0.74527 | 0.00114875 | Up-Regulated |
| SLCO4A1 | 0.74322 | 0.00963042 | Up-Regulated |
| HSPA1A | 0.73273 | 0.01680197 | Up-Regulated |
| MIR612 | 0.73094 | 0.00408443 | Up-Regulated |
| MRVI1 | 0.72730 | 0.00681676 | Up-Regulated |
| TUBB6 | 0.71172 | 0.00133786 | Up-Regulated |
| HSPB1 | 0.69598 | 0.01068947 | Up-Regulated |
| HBB | 0.67802 | 0.03317815 | Up-Regulated |
| CDKN1A | 0.66635 | 0.00093358 | Up-Regulated |
| SIK1 | 0.65139 | 0.00506942 | Up-Regulated |
| FCGBP | 0.63000 | 0.02507660 | Up-Regulated |
| NPAS4 | 0.62122 | 0.04871038 | Up-Regulated |
| ARRDC3 | 0.61433 | 0.00235475 | Up-Regulated |
| HBG1 | 0.59741 | 0.00412607 | Up-Regulated |
| MIR101-1 | -0.59042 | 0.00005341 | Down-Regulated |
| GPR34 | -0.59156 | 0.04898579 | Down-Regulated |
| FAR2 | -0.59601 | 0.00740499 | Down-Regulated |
| BC035096 | -0.60025 | 0.00645919 | Down-Regulated |
| SLC10A4 | -0.60349 | 0.01794377 | Down-Regulated |
| LOC389834 | -0.61352 | 0.03793375 | Down-Regulated |
| SYNJ1 | -0.61806 | 0.04285945 | Down-Regulated |
| CAMK2N1 | -0.62040 | 0.00152014 | Down-Regulated |
| SNAP25 | -0.62181 | 0.03099014 | Down-Regulated |
| MOXD1 | -0.63831 | 0.00195979 | Down-Regulated |
| KCNAB1 | -0.65628 | 0.00934363 | Down-Regulated |
| ND6 | -0.67624 | 0.00391936 | Down-Regulated |
| PTPN20B | -0.71188 | 0.00520405 | Down-Regulated |
| PCSK1 | -0.71687 | 0.00347985 | Down-Regulated |
| OLR1 | -0.74182 | 0.00189336 | Down-Regulated |
| TH | -0.74493 | 0.00053565 | Down-Regulated |
| BLNK | -0.75795 | 0.00125294 | Down-Regulated |
| ART3 | -0.76123 | 0.00000329 | Down-Regulated |
| P2RY13 | -0.78282 | 0.00211801 | Down-Regulated |
| ZNF385B | -0.79053 | 0.00476492 | Down-Regulated |
| ID2 | -0.79983 | 0.00229441 | Down-Regulated |
| RAB3C | -0.81313 | 0.01244976 | Down-Regulated |
| DRD1 | -0.83933 | 0.01679774 | Down-Regulated |
| GAD1 | -0.86866 | 0.02280693 | Down-Regulated |
| CX3CR1 | -0.88481 | 0.00669119 | Down-Regulated |
| P2RY12 | -1.17182 | 0.00451804 | Down-Regulated |

**Supplementary Table 6| log|FC|-value of shared genes in three brain regions**

| Gene symbol | HPC | PFC | STR |
| --- | --- | --- | --- |
| ADM | 1.2293 | 0.8635 | 0.8426 |
| HILPDA | 1.2032 | 0.6205 | 0.7983 |
| HSPB1 | 0.9258 | 0.6067 | 0.6960 |
| BAG3 | 0.7916 | 1.0237 | 0.8279 |
| DDIT4 | 1.1318 | 0.8053 | 0.8438 |
| S100A8 | 1.6463 | 1.1893 | 1.1395 |
| APOLD1 | 1.3236 | 0.8677 | 0.9635 |

**Supplementary Table 7|** **The enriched pathways related to immune system from REACTOME and KEGG database in the hippocampus between schizophrenia and control groups**

| GeneSet | Description | EnrichmentScore  （ES） | NormalizedEnrichmentScore（NES） | *P*-value | FDR | Size | LeadingEdgeNum |
| --- | --- | --- | --- | --- | --- | --- | --- |
| R-HSA-202424 | Downstream TCR signaling | -0.577076674 | -2.20897 | 0.00000 | 0.00000 | 89 | 42 |
| R-HSA-1168372 | Downstream signaling events of B Cell Receptor (BCR) | -0.614691062 | -2.25228 | 0.00000 | 0.00000 | 77 | 41 |
| R-HSA-1169091 | Activation of NF-kappaB in B cells | -0.637865376 | -2.25541 | 0.00000 | 0.00000 | 63 | 37 |
| R-HSA-2871837 | FCERI mediated NF-kB activation | -0.651565271 | -2.41151 | 0.00000 | 0.00000 | 76 | 41 |
| R-HSA-5607761 | Dectin-1 mediated noncanonical NF-kB signaling | -0.701642301 | -2.42696 | 0.00000 | 0.00000 | 56 | 37 |
| R-HSA-1236978 | Cross-presentation of soluble exogenous antigens (endosomes) | -0.740709165 | -2.46970 | 0.00000 | 0.00000 | 46 | 33 |
| R-HSA-5676590 | NIK-->noncanonical NF-kB signaling | -0.724734338 | -2.49859 | 0.00000 | 0.00000 | 55 | 37 |
| R-HSA-1236974 | ER-Phagosome pathway | -0.580397705 | -2.13724 | 0.00000 | 0.00010 | 79 | 41 |
| R-HSA-202403 | TCR signaling | -0.526745516 | -2.09371 | 0.00000 | 0.00023 | 109 | 46 |
| R-HSA-983705 | Signaling by the B Cell Receptor (BCR) | -0.5305542 | -2.05924 | 0.00000 | 0.00035 | 106 | 44 |
| R-HSA-5607764 | CLEC7A (Dectin-1) signaling | -0.542796594 | -2.06296 | 0.00000 | 0.00036 | 93 | 41 |
| R-HSA-2454202 | Fc epsilon receptor (FCERI) signaling | -0.49591633 | -1.96841 | 0.00000 | 0.00110 | 125 | 48 |
| R-HSA-1236975 | Antigen processing-Cross presentation | -0.509030621 | -1.92825 | 0.00000 | 0.00191 | 95 | 42 |
| R-HSA-6783783 | Interleukin-10 signaling | 0.609581941 | 2.19257 | 0.00000 | 0.00355 | 44 | 31 |
| R-HSA-5668541 | TNFR2 non-canonical NF-kB pathway | -0.494101149 | -1.88028 | 0.00000 | 0.00389 | 97 | 41 |
| R-HSA-6785807 | Interleukin-4 and Interleukin-13 signaling | 0.511270568 | 2.16972 | 0.00000 | 0.00462 | 107 | 33 |
| R-HSA-983168 | Antigen processing: Ubiquitination & Proteasome degradation | -0.424461824 | -1.86481 | 0.00000 | 0.00469 | 293 | 118 |
| R-HSA-5621481 | C-type lectin receptors (CLRs) | -0.451330136 | -1.79200 | 0.00000 | 0.01153 | 132 | 45 |
| R-HSA-9020702 | Interleukin-1 signaling | -0.461440646 | -1.75922 | 0.00000 | 0.01599 | 97 | 42 |
| R-HSA-5686938 | Regulation of TLR by endogenous ligand | 0.685724708 | 1.96466 | 0.00223 | 0.01755 | 18 | 4 |
| R-HSA-2029481 | FCGR activation | 0.754696048 | 1.96610 | 0.00000 | 0.01842 | 12 | 6 |
| R-HSA-6799990 | Metal sequestration by antimicrobial proteins | 0.937798424 | 1.92604 | 0.00000 | 0.02479 | 6 | 3 |
| R-HSA-983169 | Class I MHC mediated antigen processing & presentation | -0.381419859 | -1.70458 | 0.00000 | 0.02634 | 355 | 123 |
| R-HSA-909733 | Interferon alpha/beta signaling | 0.479535223 | 1.87856 | 0.00000 | 0.03768 | 67 | 22 |
| R-HSA-450341 | Activation of the AP-1 family of transcription factors | -0.706179009 | -1.65707 | 0.01239 | 0.03898 | 10 | 5 |
| R-HSA-2132295 | MHC class II antigen presentation | -0.409405413 | -1.60834 | 0.00150 | 0.05350 | 115 | 43 |
| R-HSA-5669034 | TNFs bind their physiological receptors | 0.543227682 | 1.82259 | 0.00227 | 0.06289 | 28 | 6 |
| R-HSA-168927 | TICAM1, RIP1-mediated IKK complex recruitment | -0.562188025 | -1.51616 | 0.03025 | 0.09197 | 18 | 8 |
| R-HSA-446652 | Interleukin-1 family signaling | -0.380153586 | -1.50996 | 0.00863 | 0.09535 | 132 | 44 |
| R-HSA-450282 | MAPK targets/ Nuclear events mediated by MAP kinases | -0.469449652 | -1.46594 | 0.04225 | 0.12062 | 31 | 7 |
| R-HSA-6803157 | Antimicrobial peptides | 0.428688356 | 1.70313 | 0.00276 | 0.13178 | 74 | 9 |
| R-HSA-5676594 | TNF receptor superfamily (TNFSF) members mediating non-canonical NF-kB pathway | 0.564160708 | 1.66063 | 0.01848 | 0.14342 | 18 | 9 |
| R-HSA-877300 | Interferon gamma signaling | 0.4007546 | 1.64716 | 0.00000 | 0.14852 | 87 | 39 |
| R-HSA-1280218 | Adaptive Immune System | -0.296433802 | -1.39585 | 0.00117 | 0.17253 | 720 | 195 |
| R-HSA-933542 | TRAF6 mediated NF-kB activation | 0.507788766 | 1.60239 | 0.01716 | 0.19366 | 24 | 10 |
| R-HSA-977606 | Regulation of Complement cascade | 0.44563753 | 1.57737 | 0.01463 | 0.21939 | 43 | 19 |
| R-HSA-1280215 | Cytokine Signaling in Immune system | 0.299908443 | 1.55178 | 0.00000 | 0.23205 | 653 | 121 |
| hsa04657 | IL-17 signaling pathway | 0.504308645 | 2.11182 | 0.00000 | 0.00222 | 92 | 24 |
| hsa04610 | Complement and coagulation cascades | 0.437000114 | 1.74116 | 0.00000 | 0.04234 | 77 | 32 |
| hsa04640 | Hematopoietic cell lineage | 0.375397285 | 1.56842 | 0.00279 | 0.09690 | 93 | 37 |
| hsa04062 | Chemokine signaling pathway | 0.30403064 | 1.37957 | 0.01038 | 0.15087 | 182 | 50 |
| hsa04620 | Toll-like receptor signaling pathway | 0.336831773 | 1.40523 | 0.01198 | 0.15143 | 97 | 29 |

**Supplementary Table 8| The enriched pathways related to immune system from REACTOME and KEGG database in the prefrontal cortex between schizophrenia and control groups**

| GeneSet | Description | EnrichmentScore  （ES） | NormalizedEnrichmentScore（NES） | *P*-value | FDR | Size | LeadingEdge  Num |
| --- | --- | --- | --- | --- | --- | --- | --- |
| R-HSA-6783783 | Interleukin-10 signaling | 0.66395 | 2.20803 | 0.00000 | 0.00043 | 44 | 17 |
| R-HSA-6785807 | Interleukin-4 and Interleukin-13 signaling | 0.55318 | 2.17084 | 0.00000 | 0.00173 | 107 | 35 |
| R-HSA-5686938 | Regulation of TLR by endogenous ligand | 0.77116 | 2.07260 | 0.00000 | 0.00624 | 18 | 5 |
| R-HSA-2029481 | FCGR activation | 0.81563 | 2.02773 | 0.00000 | 0.00845 | 12 | 8 |
| R-HSA-5676590 | NIK-->noncanonical NF-kB signaling | -0.53034 | -1.92924 | 0.00000 | 0.01392 | 55 | 31 |
| R-HSA-5607761 | Dectin-1 mediated noncanonical NF-kB signaling | -0.50564 | -1.83792 | 0.00000 | 0.02960 | 56 | 31 |
| R-HSA-6799990 | Metal sequestration by antimicrobial proteins | 0.93067 | 1.90502 | 0.00000 | 0.03427 | 6 | 3 |
| R-HSA-1168372 | Downstream signaling events of B Cell Receptor (BCR) | -0.45735 | -1.78821 | 0.00000 | 0.04359 | 77 | 38 |
| R-HSA-202670 | ERKs are inactivated | -0.67665 | -1.78310 | 0.00402 | 0.04456 | 13 | 4 |
| R-HSA-1169091 | Activation of NF-kappaB in B cells | -0.46354 | -1.73547 | 0.00000 | 0.06587 | 63 | 32 |
| R-HSA-977606 | Regulation of Complement cascade | 0.53509 | 1.80491 | 0.00378 | 0.07335 | 43 | 14 |
| R-HSA-6798695 | Neutrophil degranulation | 0.38061 | 1.79187 | 0.00000 | 0.07910 | 458 | 103 |
| R-HSA-2871837 | FCERI mediated NF-kB activation | -0.41920 | -1.63038 | 0.00000 | 0.12524 | 76 | 39 |
| R-HSA-166658 | Complement cascade | 0.48282 | 1.70801 | 0.00374 | 0.13400 | 54 | 14 |
| R-HSA-6803157 | Antimicrobial peptides | 0.44481 | 1.66185 | 0.00737 | 0.14473 | 74 | 21 |
| R-HSA-450282 | MAPK targets/ Nuclear events mediated by MAP kinases | -0.49183 | -1.59958 | 0.00642 | 0.14981 | 31 | 14 |
| R-HSA-1236978 | Cross-presentation of soluble exogenous antigens (endosomes) | -0.45404 | -1.60197 | 0.00864 | 0.15086 | 46 | 26 |
| R-HSA-166786 | Creation of C4 and C2 activators | 0.63385 | 1.64122 | 0.02959 | 0.15737 | 14 | 8 |
| R-HSA-198753 | ERK/MAPK targets | -0.53265 | -1.57555 | 0.02954 | 0.16734 | 22 | 9 |
| R-HSA-1280215 | Cytokine Signaling in Immune system | 0.33450 | 1.61682 | 0.00000 | 0.17619 | 653 | 144 |
| R-HSA-912631 | Regulation of signaling by CBL | -0.52601 | -1.55639 | 0.01695 | 0.18089 | 22 | 4 |
| R-HSA-933542 | TRAF6 mediated NF-kB activation | 0.55058 | 1.59106 | 0.03327 | 0.18391 | 24 | 10 |
| R-HSA-449147 | Signaling by Interleukins | 0.33272 | 1.54798 | 0.00000 | 0.22589 | 438 | 76 |
| R-HSA-1169092 | Activation of RAS in B cells | -0.74931 | -1.48341 | 0.04815 | 0.23871 | 5 | 2 |
| hsa04657 | IL-17 signaling pathway | 0.50800 | 1.98709 | 0.00000 | 0.00335 | 92 | 22 |
| hsa04610 | Complement and coagulation cascades | 0.45144 | 1.69155 | 0.00169 | 0.06142 | 77 | 19 |
| hsa04612 | Antigen processing and presentation | 0.46078 | 1.66403 | 0.00363 | 0.06142 | 70 | 24 |
| hsa04620 | Toll-like receptor signaling pathway | 0.40681 | 1.59028 | 0.01068 | 0.07917 | 97 | 33 |
| hsa04623 | Cytosolic DNA-sensing pathway | 0.44170 | 1.57250 | 0.01757 | 0.08569 | 57 | 20 |
| hsa04640 | Hematopoietic cell lineage | 0.35786 | 1.39271 | 0.03885 | 0.20205 | 93 | 28 |
| hsa04659 | Th17 cell differentiation | 0.34919 | 1.37971 | 0.04303 | 0.21268 | 103 | 38 |

**Supplementary Table 9| The enriched pathways related to immune system from REACTOME and KEGG database in the striatum between schizophrenia and control groups**

| GeneSet | Description | EnrichmentScore  （ES） | NormalizedEnrichmentScore(NES) | *P*-value | FDR | Size | LeadingEdge  Num |
| --- | --- | --- | --- | --- | --- | --- | --- |
| R-HSA-6785807 | Interleukin-4 and Interleukin-13 signaling | 0.55691 | 2.29039 | 0.00000 | 0.00000 | 107 | 31 |
| R-HSA-5676590 | NIK-->noncanonical NF-kB signaling | -0.56733 | -1.94764 | 0.00161 | 0.00373 | 55 | 30 |
| R-HSA-1168372 | Downstream signaling events of B Cell Receptor (BCR) | -0.52995 | -1.91147 | 0.00000 | 0.00501 | 77 | 38 |
| R-HSA-1236978 | Cross-presentation of soluble exogenous antigens (endosomes) | -0.57520 | -1.90623 | 0.00000 | 0.00549 | 46 | 25 |
| R-HSA-983705 | Signaling by the B Cell Receptor (BCR) | -0.49395 | -1.86885 | 0.00000 | 0.00824 | 106 | 46 |
| R-HSA-5607761 | Dectin-1 mediated noncanonical NF-kB signaling | -0.53870 | -1.84934 | 0.00161 | 0.00991 | 56 | 30 |
| R-HSA-5607764 | CLEC7A (Dectin-1) signaling | -0.48805 | -1.81688 | 0.00000 | 0.01354 | 93 | 46 |
| R-HSA-6783783 | Interleukin-10 signaling | 0.57842 | 1.99369 | 0.00000 | 0.01466 | 44 | 13 |
| R-HSA-1169091 | Activation of NF-kappaB in B cells | -0.50501 | -1.75009 | 0.00161 | 0.02422 | 63 | 31 |
| R-HSA-2871837 | FCERI mediated NF-kB activation | -0.48612 | -1.74680 | 0.00000 | 0.02486 | 76 | 36 |
| R-HSA-202424 | Downstream TCR signaling | -0.46001 | -1.69439 | 0.00000 | 0.03998 | 89 | 37 |
| R-HSA-168927 | TICAM1, RIP1-mediated IKK complex recruitment | -0.61991 | -1.66036 | 0.01085 | 0.04966 | 18 | 7 |
| R-HSA-909733 | Interferon alpha/beta signaling | 0.49067 | 1.83205 | 0.00000 | 0.05613 | 67 | 29 |
| R-HSA-2025928 | Calcineurin activates NFAT | -0.71704 | -1.62311 | 0.02747 | 0.06437 | 9 | 5 |
| R-HSA-5684264 | MAP3K8 (TPL2)-dependent MAPK1/3 activation | -0.60058 | -1.56985 | 0.03004 | 0.09070 | 16 | 9 |
| R-HSA-6799990 | Metal sequestration by antimicrobial proteins | 0.84246 | 1.70868 | 0.00000 | 0.10240 | 6 | 3 |
| R-HSA-202430 | Translocation of ZAP-70 to Immunological synapse | -0.59118 | -1.54143 | 0.03978 | 0.10295 | 16 | 2 |
| R-HSA-983168 | Antigen processing: Ubiquitination & Proteasome degradation | -0.35982 | -1.53654 | 0.00000 | 0.10507 | 293 | 103 |
| R-HSA-5621481 | C-type lectin receptors (CLRs) | -0.38454 | -1.51435 | 0.00709 | 0.11551 | 132 | 51 |
| R-HSA-6803157 | Antimicrobial peptides | 0.42774 | 1.65223 | 0.00267 | 0.12631 | 74 | 20 |
| R-HSA-2454202 | Fc epsilon receptor (FCERI) signaling | -0.39072 | -1.49703 | 0.00448 | 0.12669 | 125 | 52 |
| R-HSA-202403 | TCR signaling | -0.39501 | -1.49863 | 0.00769 | 0.12706 | 109 | 41 |
| R-HSA-5686938 | Regulation of TLR by endogenous ligand | 0.60144 | 1.63962 | 0.01762 | 0.13647 | 18 | 2 |
| R-HSA-450294 | MAP kinase activation | -0.41778 | -1.44893 | 0.02899 | 0.15517 | 61 | 24 |
| R-HSA-166658 | Complement cascade | 0.44188 | 1.57756 | 0.01542 | 0.19354 | 54 | 10 |
